# Synthesis and Characterization of New Dihydronaphthalene Candidates as Potent Cytotoxic Agents against MCF-7 Human Cancer Cells

**DOI:** 10.1155/2020/8649745

**Published:** 2020-12-23

**Authors:** Nesreen S. Ahmed, Alaadin E. Sarhan, Aisha A. K. Al-Ashmawy, Abd El-Galil E. Amr, Mogedda E. Haiba, Elsayed A. Elsayed

**Affiliations:** ^1^Department of Therapeutic Chemistry, Pharmaceutical and Drug Industries Research Division, National Research Center, El Buhouth Street, Dokki, Cairo 12622, Egypt; ^2^Pharmaceutical Chemistry Department, College of Pharmacy, King Saud University, Riyadh 11451, Saudi Arabia; ^3^Applied Organic Chemistry Department, National Research Center, Cairo, Dokki 12622, Egypt; ^4^Zoology Department, Faculty of Science, King Saud University, Riyadh 11451, Saudi Arabia; ^5^Chemistry of Natural and Microbial Products Department, National Research Centre, Dokki, 12622 Cairo, Egypt

## Abstract

In the present work, a new series of dihydronaphthalene derivatives were synthesized starting with 6-methoxy-1-tetralone **1**, and the corresponding hydrazine derivative **2**. Reaction of compound **2** with aryl isothiocyanates produced thiosemicarbazides **3a-d**, which were reacted with ethyl chloroacetate to give thiazolidinone derivatives **4a-d**. Pyrano thiazolecarbonitrile derivatives **5a**-**f** were prepared by heating a mixture of compounds **4a** or **4c**, aryl aldehydes, and malononitrile utilizing distilled water in the presence of catalytic amount of potassium hydrogen phthalate. Also, treatment of **4a** with DMF-DMA under solvent-free conditions gave enaminone derivative **6**, which condensed with ethyl acetoacetate or acetylacetone or malononitrile or cyanothioacetamide to give compounds **7**-**10**, respectively. Finally, reaction of the enaminone **6** with 2-aminoimidazol or 2-aminothiazol in the presence of glacial acetic acid produced derivatives **11** and **12**, respectively. Cytotoxic evaluation of eleven compounds, against MCF-7 (human breast adenocarcinoma) cell lines, was estimated. Results revealed that five of the examined compounds **5a**, **5d**, **5e**, **10**, and **3d** showed potent cytotoxic activities recording, IC_50_ values; 0.93 ± 0.02, 1.76 ± 0.04, 2.36 ± 0.06, 2.83 ± 0.07, and 3.73 ± 0.09 *μ*M, respectively, which were more potent than the reference used (Saturosporin, IC_50_6.08 ± 0.15 *μ*M). The new products were also examined towards normal epithelial breast cells (MCF10A). All of them showed very good safety profile with different degrees and were safer than the reference drug used. Compound **5a** was the most effective against MCF-7 cells and was less toxic than Saturosporin by about 18.45-folds towards MCF01A normal cells. All the new compounds were fully characterized by the different spectral and analytical tools. Herein, detailed syntheses, spectroscopic, and biological data are reported.

## 1. Introduction

Breast cancer represents the most common cancer disease among women. It represents the second-highest rate leading cause of women mortality worldwide [1]. Chemotherapy is the most common for cancer treatment. Developing cancer drugs is essential to discover more active products with high potential [2]. This work represents an attempt to develop new therapeutic compounds of high efficacy in treating breast cancer disease. Literature reports confirmed the important diverse types of pharmaceutical activities of thiazole, pyrane, and/or pyridine derivatives. Thiazoles have concerned a great era of attention due to their association with various types of biological activities. Their derivatives exhibited important potency as anticancer [3–5], antibacterial [6], anti-inflammatory [7], antioxidant [8], antimalarial [9] agents, and HIV-inhibitors [10]. Also, pyrane containing derivatives have been identified as anticancer [11], antimicrobial [12], anti-inflammatory [13], and antimalarial [14] agents. Furthermore, literature survey reported that compounds containing pyridine ring demonstrated anticancer [15], antimicrobial [16], anti-inflammatory [17], antiviral [18], and antioxidant [19] activities. Based on our recent work which demonstrated that various tetralone-based derivatives verified significant inhibitory activity towards different types of cancer disease, they displayed highly significant cytotoxic activity against MCF-7 human cancer cells (breast cancer), for all the tested compounds, all of them showed activity more potent than that obtained by the reference drug (Doxorubicine) [20]. Also, significant cytotoxic effects on U373 (human glioblastoma cells) were testified [21]. Additionally, cytotoxic activity against HepG-2 (human cancer cells) was established [22]. These facts motivated us to synthesize new compounds derived from coupling of 6-methoxy-1-tetralone with different heterocyclic ring systems such as thiazole, pyrano [2,3-d] thiazole, and dihydrothiazolo [4,5-b] pyridine in one molecule aiming to construct new candidates of enhancing anticancer activity. Herein, 6-methoxy-1-tetralone was used as good building blocks to construct the desired heterocyclic products.

## 2. Materials and Methods

### 2.1. Chemistry

Melting points were uncorrected and were taken in open capillary tubes using Electrothermal apparatus 9100. Infrared spectra were recorded on a Shimadzu FT-IR Affinity-1 Spectrometer, Infrared spectrometer at cm^−1^ scale using KBr disc technique at Faculty of Pharmacy-Cairo University, Cairo, Egypt. ^1^H NMR and ^13^C NMR spectra were determined by using a Bruker High-Performance Digital FT-NMR Spectrometer Avance III 400 MHz, Faculty of Pharmacy-Cairo University, Cairo, Egypt. Chemical shifts were expressed in *δ* (ppm) downfield from TMS as an internal standard. The mass spectra were recorded on a GCMC-QP 1000 EX Shimadzu gas chromatograph-mass spectrometer (GC-MS; Shimadzu Corp. Kyoto, Japan) at electron ionization (EI) of 70 eV. Elemental analyses (C, H, and N) were conducted at the Micro Analytical Center of the Faculty of Science of Cairo University, Cairo, Egypt. All reagents were commercial grade and used without further purification. Reaction progress was monitored by thin-layer chromatography (TLC) on precoated (0.75 mm) silica gel GF254 plates (Merck Group, Darmstadt, Germany). Products were visualized under ultraviolet (UV) light.

#### 2.1.1. Synthesis of 6-Methoxy-3, 4-dihydronaphthalen-1(*2H*)-ylidene hydrazine 2

Compound **2** was prepared and characterized as described in our literature report [23].

#### 2.1.2. N-(Substituted)-2-(6-methoxy-3,4-dihydronaphthalen-1(*2H*)-ylidene) hydrazinecarbothio amide (3a-d)

A mixture of compound **2** (0.002 mol) and the appropriate substituted isothiocyanates (0.002 mol) namely (p-bromophenyl, p-flourophenyl, phenoxyphenyl, or ethyl) isothiocyanate in dry benzene (30 mL) was refluxed for 30 min. After cooling, the excess solvent was evaporated; the solid product was filtered off, dried, and recrystallized from ethyl alcohol to give the desired products **3a**-**d**, respectively.

#### 2.1.3. N-(4-Bromophenyl)-2-(6-methoxy-3,4-dihydronaphthalen-1(*2H*)-ylidene)hydrazine-1-carbo thioamide 3a

Yield: 91%; m.p.: 191-193°C; IR (*υ*_max_/cm^−1^): 3381, 3317 (2NH); ^1^H-NMR (DMSO-*d_6_*): *δ*, 1.82-1.85 (m, 2H, CH_2_, C-3), 2.58 (t, *J* =1.7, 2H, CH_2_, C-2), 2.7 (t, *J* =6.5, 2H, CH_2_, C-4), 3.79 (s, 3H, OCH_3_), 6.7-6.8 (m, 2H, Ar), 7.5-7.6 (m, 4H, Ar), 8.3 (d, *J* =8.6, 1H, Ar, CH-8), 10.0, 10.5 (2 s, 2H, 2NH); ^13^C-NMR (DMSO-*d_6_*): *δ*; 21.9 (CH_2_, C-3), 26.5 (CH2, C-2), 29.6 (CH_2_,C-4), 55.6 (OCH_3_), 112.7, 113.6, 117.8, 124.8, 128.0, 128.1, 131.2, 139.1, 142.8, 150.2, 160.7 (Ar-C and CN), 176.8 (C=S); MS: m/z (%) 403, 405 (M^+^, 37, 36) consistent with the molecular formula C_18_H_18_Br N_3_OS. Anal. Calcd. C,53.47; H,4.49; N,10.39, Found C,53.69; H, 3.99; N,10.71%.

#### 2.1.4. N-(4-Fluorophenyl)-2-(6-methoxy-3,4-dihydronaphthalen-1(*2H*)-ylidene)hydrazine-1-carbo thioamide 3b

Yield: 85%; m.p.: 154-156°C; IR (*υ*_max_/cm^−1^): 3365, 3320 (2NH); ^1^H-NMR (DMSO-*d_6_*): *δ*, 1.8 (m, 2H, CH_2,_ C-3), 2.6 (t, *J* = 6, 2H, CH_2_, C-2), 2.9 (t, *J* = 6, 2H, CH_2_, C-4), 3.8 (s, 3H, OCH_3_), 6.7 (s, 1H, Ar, CH-5), 7.2-7.5 (m, 4H, Ar), 7.68 (d, *J* = 8.5, 1H, Ar), 8.2 (d, *J* = 8.5, 1H, Ar), 8.3 (br s, 1H, NH), 9.3 (br s, 1H, NH); ^13^C-NMR (DMSO-*d_6_*): *δ*, 20.5 (CH_2_, C-3), 26.5 (CH_2_,C-2), 27.9 (CH_2_, C-4), 55.6 (OCH_3_), 111.2, 113.6, 119.4, 120.8, 123.4, 124.4, 126.4, 127.1, 129.8, 131.1, 133.2, 134.1, 158.2, 159.1, 168.5, (Ar-C and CN), 170.3 (C=S); MS: m/z (%) 343 (M^+^, 25) consistent with the molecular formula C_18_H_18_FN_3_OS. Anal. Calcd. C, 62.95; H, 5.28; N, 12.24,Found C,62.53; H, 4.73; N,11.81%.

#### 2.1.5. 2-(6-Methoxy-3,4-dihydronaphthalen-1(*2H*)-ylidene)-N-(4-phenoxyphenyl)-hydrazine-1-carbo thioamide 3c

Yield: 94%; m.p.: 171°C; IR (*υ*_max_/cm^−1^): 3325, 3275 (2NH); ^1^H-NMR (DMSO-*d_6_*): *δ*, 1.85 (m, 2H, CH_2_-3), 2.7 (t, *J* =6.5, 2H, CH_2_-2), 2.86 (t, *J* = 6, 2H, CH_2_-4), 3.8 (s, 3H, OCH_3_), 6.2 (t, *J* =6.5, 1H, C-4`, phenyl ring), 6.78 (s, 1H, Ar, CH-5), 7.1-7.3 (m, 8H, Ar and NH), 7.65 (d, *J* = 8.5, 1H, Ar), 8.4 (d, *J* = 8.5, 1H, Ar), 8.6 (br s, 1H, NH); ^13^C-NMR (DMSO-*d_6_*): *δ*, 20.5 (CH_2_-3), 27.9 (CH_2_-2), 29.5 (CH_2_-4), 55.6 (OCH_3_), 111.2, 113.6, 119.4, 120.8, 123.4, 124.4, 126.4, 127.1, 129.8, 130.0, 133.2, 134.1, 158.2, 159.1, 168.5, (Ar-C and CN), 170.3 (C=S); MS: m/z (%) 417 (M^+^, 53) consistent with the molecular formula C_24_H_23_N_3_O_2_S. Anal. Calcd. C, 69.04; H, 5.55; N, 10.06, Found C,68.67; H, 5.16; N,9.68%.

#### 2.1.6. N-(4-Ethyl)-2-(6-methoxy-3,4-dihydronaphthalen-1(*2H*)-ylidene)hydrazine-1-carbothioamide 3d

Yield: 93%; m.p.: 150-153°C; IR (*υ*_max_/cm^−1^): 3370, 3281 (2NH); ^1^H-NMR (DMSO-*d_6_*): *δ*,1.1 (t, *J* = 5.9, 3H, -CH_2_CH_3_), 1.79 (m, 2H, CH_2_, C-3), 2.6 (t, *J* = 6.4, 2H, CH_2_, C-2), 2.7 (t, *J* = 5.8, 2H, CH_2_, C-4), 3.59-3.64 (q, 2H, -CH_2_-CH_3_), 3.79 (s, 3H, OCH_3_), 6.7 (s, 1H, CH-5), 6.8 (d, *J* = 8.8, 1H, CH-7), 8.2 (d, *J* = 8.8, 1H, CH-8), 8.4 (br s, 1H, NH), 9.9 (s, 1H, NH); ^13^C-NMR (DMSO-*d_6_*): *δ*,14.8 (CH_2_CH_3_), 22.3 (CH_2_-3), 27.01 (CH_2_-2), 29.6 (CH_2_-4), 38.8 (CH_2_CH_3_), 55.5 (OCH_3_), 112.7, 113.5, 125.9, 128.7, 142.6, 148.3, 160.7 (Ar-C and CN), (177 C=S); MS: m/z (%)277 (M^+^, 65) consistent with the molecular formula C_14_H_19_N_3_OS. Anal. Calcd. C, 60.62; H, 6.90; N, 15.15; Found C, 60.13; H, 6.63; N, 14.98%.

#### 2.1.7. 2-Substituted-3-((6-methoxy-3,4-dihydronaphthalin-1(*2H*)-ylidene)amino)thiazolidin-4-ones 4a-d

To a solution of **3a**-**d** (0.001 mol) in ethanol (20 mL), ethyl chloroacetate (0.001 mol) was added and refluxed for 4 h. After cooling, the solid product was filtered off, dried, and recrystallized from ethanol to give the desired compounds **4a**-**d**.

#### 2.1.8. 2-((4-Bromophenyl)imino)-3-((6-methoxy-3,4-dihydronaphthalen-1(*2H*)-ylidene)amino) thiazolidin-4-one 4a

Yield: 81%; m.p.: 183°C; IR (*υ*_max_/cm^−1^): 1710 (C=O); ^1^H-NMR (DMSO-*d_6_*): *δ*, 1.8 (m, 2H, CH-3), 2.55 (t, *J* = 6.4, 2H, CH_2_-2), 2.7 (t, *J* = 6, 2H, CH_2_-4), 3.7 (s, 3H, OCH_3_), 4.05 (s, 2H, CH_2_ thiazolidinone), 6.7-6.8 (m, 3H, Ar), 7.3 (d, *J* = 8.5, 1H, Ar), 7.5-7.6 (m, 2H, Ar), 8.3 (d, *J* = 8.5, 1H, Ar, CH-8); ^13^C-NMR (DMSO-*d_6_*): *δ*; 22.5 (CH_2_, C-3), 26.8 (CH_2_, C-2), 29.7 (CH_2_,C-4), 32.9 (CH_2_, thizolidinone), 55.6 (OCH_3_), 110.7, 112.7, 113.6, 117.8, 124.8, 128.0, 128.1, 131.2, 139.1, 142.8, 150.2, 160.7, 167.4 (Ar-C, CN and C=O); MS: m/z (%): 443, 445 (M^+^, 5, 5) consistent with the molecular formula C_20_H_18_ BrN_3_O_2_S. Anal. Calcd. C, 54.06; H, 4.08; N, 9.46; Found C, 53.61; H, 3.79; N,8.91%.

#### 2.1.9. 2-((4-Fluorophenyl)imino)-3-((6-methoxy-3,4-dihydronaphthalen-1(*2H*)-ylidene)amino) thiazolidin-4-one 4b

Yield: 89%; m.p.: 190°C; IR (*υ*_max_/cm^−1^): 1728 (C=O); ^1^H-NMR (DMSO-*d_6_*): *δ*, 1.9 (m, 2H, CH_2_-3), 2.7 (t, *J* = 5.9, 2H, CH_2_-2), 2.9 (t, *J* =6.5, 2H, CH_2_-4), 3.8 (s, 3H, OCH_3_), 4.06 (s, 2H, thiazolidinone), 6.6 (s, 1H, Ar, CH-5), 6.8-6.9 (m, 3H, Ar), 7.1-7.4 (m, 2H, Ar), 8.3 (d, *J* = 9.3, 1H, Ar, CH-8); ^13^C-NMR (DMSO-*d_6_*): *δ*, 21.4 (CH_2_-3), 27.4 (CH_2_-2), 29.6 (CH_2_-4), 32.7 CH_2_ thiazolidinone), 55.3 (OCH_3_), 111.03, 112.9, 113.2, 113.4, 114.2, 116.2, 116.8, 124.2, 125.5, 128.1, 129.6, 130.5, 143.4, 146.9, 161.7, 162.9 (Ar-C and CN), 171 (C=O); MS: m/z (%) 383, (M^+^, 1) consistent with the molecular formula C_20_H_18_FN_3_O_2_S. Anal. Calcd. C, 62.65; H, 4.73; N, 10.96; Found C, 62.34; H, 7.53; N,10.75%.

#### 2.1.10. 3-((6-Methoxy-3,4-dihydronaphthalen-1(*2H*)-ylidene)amino)-2-((4-phenoxyphenyl)imino) thiazolidin-4-one 4c

Yield: 94%; m.p.: 171°C; IR (*υ*_max_/cm^−1^): 1717 (C=O); ^1^H-NMR (DMSO-*d_6_*): *δ*, 1.86 (m, 2H, CH_2_-3), 2.7 (t, *J* =6.5, 2H, CH_2_-2), 2.8 (t, *J* = 6, 2H, CH_2_-4), 3.8 (s, 3H, OCH_3_), 3.99 (s, 2H, CH_2_ thiazolidinone), 6.2 (t, *J* =6.5, 1H, C-4, phenyl ring), 6.7 (s, 1H, Ar, CH-5), 7.1-7.3 (m, 7H, Ar), 7.6 (d, *J* = 8.5, 2H, Ar), 8.3 (d, *J* = 8.5, 1H, Ar); ^13^C-NMR (DMSO-*d_6_*): *δ*; 20.5 (CH_2_-3), 27.9 (CH_2_-2), 29.5 (CH_2_-4), 32.6 (CH_2_ thiazolidinone), 55.6 (OCH_3_), 111.2, 113.6, 119.4, 120.8, 123.4, 124.4, 126.4, 127.1, 129.8, 130.0, 133.2, 134.1, 145.8, 158.2, 159.1, 160.6, (Ar-C and CN), 177.1 C=O); MS: m/z (%) 457 (M^+^, 67) consistent with the molecular formula C_26_H_23_N_3_O_3_S. Anal. Calcd. C, 68.25; H, 5.07; N, 9.18; Found C, 69.12; H, 4.62; N,8.71%.

#### 2.1.11. 2-(Ethylimino)-3-((6-methoxy-3,4-dihydronaphthalen-1(*2H*)-ylidene)amino)thiazolidin-4-one 4d

Yield: 76%; m.p.: 117°C; IR (*υ*_max_/cm^−1^): 1707 (C=O); ^1^H-NMR (DMSO-*d_6_*): *δ*, 1.2 (t, *J* = 7, 3H, -CH_2_-CH_3_), 1.7-1.8 (m, 2H, CH_2_-3), 2.7 (t, *J* = 5.8, 2H, CH_2_-2), 2.8 (t, *J* =6.5, 2H, CH_2_-4), 3.74-3.79 (m, 5H, OCH_3_ and –CH_2_CH_3_), 3.9 (s, 2H, CH_2_ thiazolidinone), 6.7 (s, 1H, Ar, CH-5), 6.8 (d, *J* = 8.8, 1H, Ar, CH-7), 8.05 (d, *J* = 8.8, 1H, Ar, CH-8); ^13^C-NMR (DMSO-*d_6_*): *δ*, 12.6 (CH_2_CH_3_), 22.2 (CH_2_-3), 27.1 (CH_2_-2), 29.9 (CH_2_-4), 32.4 (CH_2_ thiazolidinone), 38.2 (CH_2_CH_3_), 55.6 (OCH_3_), 113.06, 113.6, 125.2, 126.8, 143.1, 161.0, 161.1, 161.3, (Ar-C and CN), 172.3, (C=O); MS: m/z (%): 317 (M^+^, 68) consistent with the molecular formula C_16_H_19_N_3_O_2_S. Anal. Calcd. C, 60.55; H, 6.03; N, 13.24; Found C, 60.09; H, 5.74; N, 12.88%.

#### 2.1.12. 5-Amino-2-(4-substitutedimino)-3-((6-methoxy-3,4-dihydronaphthalen-1(*2H*)-ylidene) amino)-7-(4-substitutedphenyl)-3,7-dihydro-2H-pyrano [2,3-d]thiazole-6-carbonitrile 5a-f

To a mixture of thiazolidinone derivatives **4a** or **4c** (0.001 mol), aryl aldehyde, namely, 4-methoxy benzaldehyde, 4-nitrobenzaldehyde and 2-furaldehyde (0.001 mole), and malononitrile (0.001 mole), potassium hydrogen phthalate (KHP) (25 mol %) in distilled water (5 mL), was added. The mixture was heated at 50°C, after completion of the reaction and cooling, and the solid product was collected by filtration, washed with distilled water, dried, and recrystallized from dilute ethanol to give compounds (**5a-f**), respectively.

#### 2.1.13. 5-Amino-2-((4-bromophenyl)imino)-3-((6-methoxy-3,4-dihydronaphthalen-1(*2H*)-ylidene) amino)-7-(4-methoxyphenyl)-3,7-dihydro-2H-pyrano [2,3-d]thiazole-6-carbonitrile 5a

Yield: 78%; m.p.: 247°C; IR (*υ*_max_/cm^−1^): 3470, 3366 (NH_2_), 2208 (CN); ^1^H-NMR (DMSO-*d_6_*): *δ*, 1.7 (m, 2H, CH_2_-3), 2.57 (t, *J* = 6.2, 2H, CH_2_-2), 2.73 (t, *J* = 5.7, 2H, CH_2_-4), 3.74 (s, 6H, 2OCH_3_), 3.78 (s, 1H, CH-4, pyran), 4.06 (s, 2H, NH_2_), 6.7 (s, 1H, Ar, H-5), 6.8 (d, *J* = 8.8, 1H, Ar), 7.09-7.23 (m, 4H, Ar), 7.40-7.5 (m, 4H, Ar), 8.05 (d, *J* = 8.8, 1H, Ar, H-8). ^13^C-NMR (DMSO-*d_6_*): *δ*, 22.2 (CH_2_-3), 27.1 (CH_2_-2), 29.6 (CH_2_-4), 32.6 (CH-4, pyran), 45.7 (C-5, pyran), 55.7 (2OCH_3_), 65.7 (C-3, pyran), 113.0, 113.76, 118.6, 119.8, 124.6, 125.2, 126.9, 130.1, 130.4, 130.7, 143.0, 156.3, 157.2, 161.1, 161.5, 162.1, 165.9, 168.1, 169.0 (Ar-C and CN); MS: m/z (%): 628.630 (M^+^, 100, 98) consistent with the molecular formula C_31_H_26_BrN_5_O_3_S. Anal. Calcd. C, 59.24; H, 4.17; N, 11.14; Found C, 60.23; H, 3.71; N, 10.96%.

#### 2.1.14. 5-Amino-2-((4-bromophenyl)imino)-3-((6-methoxy-3,4-dihydronaphthalen-1(*2H*)-ylidene) amino)-7-(4-nitrophenyl)-3,7-dihydro-2H-pyrano [2,3-d]thiazole-6-carbonitrile 5b

Yield: 56%; m.p.: 213°C; IR (*υ*_max_/cm^−1^): 3450, 3373 (NH_2_), 2195 (CN); ^1^H-NMR (DMSO-*d_6_*): *δ*, 1.9 (m, 2H, CH_2_-3), 2.7 (t, *J* = 6.2, 2H, CH_2_-2), 2.9 (t, *J* = 5.7, 2H, CH_2_-4), 3.8 (s, 3H, OCH_3_), 4.0 (s, 1H, CH-4, pyran), 6.7 (s, 1H, Ar, H-5), 7.4-7.9 (m, 11H, Ar and NH_2_), 8.3 (d, *J* = 8.2, 1H, Ar, H-8).^13^C-NMR (DMSO-*d_6_*): *δ*, 22.3 (CH_2_-3), 27.4 (CH_2_-2), 29.9 (CH_2_-4), 31.7 (CH-4, pyran), 45.5 (CH-5, pyran), 55.67 (OCH_3_), 67.8 (C-3, pyran), 113.0, 113.76, 118.6, 119.8, 124.6, 125.2, 126.9, 130.4, 130.7, 140.7, 143.0, 146.0, 156.3, 159.5, 161.1, 161.5, 162.1, 165.9, 168.1 (Ar-C and CN); MS: m/z (%): 641, 642 (M^+^, 15 13.9) consistent with the molecular formula C_30_H_23_BrN_6_O_4_S. Anal. Calcd. C, 55.99; H, 3.60; N, 13.06; Found C, 55.39; H, 3.41; N, 12.81%.

#### 2.1.15. 5-Amino-2-((4-bromophenyl)imino)-7-(4-furan-2-yl)-3-((6-methoxy-3,4-dihydronaphthalen -1(*2H*)-ylidene)amino)-3,7-dihydro-2H-pyrano [2,3-d]thiazole-6-carbonitrile 5c

Yield: 72%; m.p.: 255°C; IR (*υ*_max_/cm^−1^): 3428, 3310 (NH_2_), 2210 (CN); ^1^H-NMR (DMSO-*d_6_*): *δ*, 1.7 (m, 2H, CH_2_-3), 2.57 (t, *J* =6.5, 2H, CH_2_-2), 2.7 (t, *J* = 5.7, 2H, CH_2_-4), 3.7 (s, 3H, OCH_3_), 4.05 (s, H, CH-4, pyran), 5.06 (s, 2H, NH_2_), 6.7 (s, 1H, Ar, H-5), 6.8 (t, *J* = 5.9, 1H, furyl), 7.0-7.5 (m, 5H, Ar), 8.0 (d, *J* = 9.3, 1H, Ar), 8.1 (d, *J* = 9.3, 1H, Ar), 8.3 (d, *J* = 8.5, 1H, Ar, H-8); ^13^C-NMR (DMSO-*d_6_*): *δ*, 22.2 (CH_2_-3), 27.1 (CH_2_-2), 29.7 (CH_2_-4), 32.6 (CH-4, pyran), 45.7 (C-5, pyran), 55.6 (OCH_3_), 67.9 (C-3, pyran), 106.2, 107.5, 110.8, 118.6, 119.8, 124.6, 125.2, 130.1, 130.7, 143.0, 146.3, 156.3, 157.2, 161.1, 161.5, 162.1, 165.9, 168.1, 169.0 (Ar-C and CN); MS: m/z (%): 587, 590 (M^+^, 59, 58) consistent with the molecular formula C_28_H_22_BrN_5_O_3_S. Anal. Calcd. C, 57.15; H, 3.77; N, 11.90; Found C, 56.87; H, 3.63; N, 11.48%.

#### 2.1.16. 5-Amino-3-((6-methoxy-3,4-dihydronaphthalen-1(*2H*)-ylidene)amino)-7-(4-methoxyphenyl) -2-((4-phenoxyphenyl)imino)--3,7-dihydro-2H-pyrano [2,3-d]thiazole-6-carbonitrile 5d

Yield: 81%; m.p.: 209°C; IR (*υ*_max_/cm^−1^): 3444, 3370 (NH_2_), 2215 (CN); ^1^H-NMR (DMSO-*d_6_*): *δ*, 1.7 (m, 2H, CH_2_-3), 2.4 (t, *J* = 6.2, 2H, CH_2_-2), 2.7 (t, *J* = 5.7, 2H, CH_2_-4), 3.72 (s, 3H, OCH_3_), 3.78 (s, 3H, OCH_3_), 4.05 (s, 1H, CH-4, pyran), 6.7 (s, 1H, Ar, H-5), 6.8-7.3 (m, 10H, Ar), 7.4 (d, *J* = 7.6, 2H, Ar), 8.02 (d, *J* = 8.5, 2H, Ar), 8.1 (d, *J* = 8.5, 1H, Ar, H-8), 10.5 (s, 2H, NH_2_); ^13^C-NMR (DMSO-*d_6_*): *δ*, 21.9 (CH_2_-3), 26.5 (CH_2_-2), 29.6 (CH_2_-4), 32.6 (CH-4, pyran), 45.7 (C-5, pyran), 55.64, 55.66 (2OCH_3_), 65.9 (C-3, pyran), 113.0, 113.76, 114.8, 117.2, 118.6, 119.8, 120.9, 124.6, 125.2, 126.9, 130.1, 130.4, 131.2, 134.1, 143.0, 156.3, 157.2, 161.1, 161.5, 162.1, 165.9, 168.1, 169.0 (Ar-C and CN); MS: m/z (%): 641 (M^+^, 42) consistent with the molecular formula C_37_ H_31_N_5_O_4_S. Anal. Calcd. C, 69.25; H, 4.87; N, 10.90; Found C, 69.09; H, 4.63; N, 10.67%.

#### 2.1.17. 5-Amino-3-((6-methoxy-3,4-dihydronaphthalen-1(*2H*)-ylidene)amino)-7-(4-nitrophenyl)-2-((4-phenoxyphenyl)imino)--3,7-dihydro-2H-pyrano [2,3-d]thiazole-6-carbonitrile 5e

Yield: 61%; m.p.: 223°C; IR (*υ*_max_/cm^−1^): 3465, 3360 (NH_2_), 2197 (CN); ^1^H-NMR (DMSO-*d_6_*): *δ*, 1.7 (m, 2H, CH_2_-3), 1.8 (t, *J* = 6.2, 2H, CH_2_-2), 2.9 (t, *J* = 5.7, 2H, CH_2_-4), 3.8 (s, 3H, OCH_3_), 4.05 (s, 1H, CH-4, pyran), 6.7 (s, 1H, Ar, H-5), 6.8-7.4 (m, 10H, Ar), 7.53 (d, *J* = 9.3, 2H, Ar), 7.6 (d, *J* = 9.3, 2H, Ar), 8.2 (d, *J* = 8.5, 1H, Ar, H-8), 12.2 (s, 2H, NH_2_); ^13^C-NMR (DMSO-*d_6_*): *δ*, 21.9 (CH_2_-3), 27.1 (CH_2_-2), 29.9 (CH_2_-4), 31.6 (CH-4, pyran), 46.0 (C-5, pyran), 55.2 (OCH_3_), 66.8 (C-3, pyran), 113.0, 113.7, 118.6, 119.8, 124.6, 125.2, 126.9, 130.1, 130.4, 130.7, 142.7, 143.0, 144.9, 149.0, 153.8, 156.3, 157.2, 161.1, 161.5, 162.1, 165.9, 168.1, 169.0 (Ar-C and CN); MS: m/z (%): 656 (M^+^, 63) consistent with the molecular formula C_36_ H_28_N_6_O_5_S. Anal. Calcd. C, 65.84; H, 4.30; N, 12.80; Found C, 66.08; H, 4.11; N, 12.68%.

#### 2.1.18. 5-Amino-7-(furan-2-yl)-3-((6-methoxy-3,4-dihydronaphthalen-1(*2H*)-ylidene)amino)-2-((4-phenoxyphenyl)imino)- 3,7-dihydro-2H-pyrano [2,3-d]thiazole-6-carbonitrile 5f

Yield: 55%; m.p.: 280°C; IR (*υ*_max_/cm^−1^): 3475, 3334 (NH_2_), 2208 (CN); ^1^H-NMR (DMSO-*d_6_*): *δ*, 1.9 (m, 2H, CH_2_-3), 2.4 (t, *J* = 5.9, 2H, CH_2_-2), 2.9 (t, *J* = 5.7, 2H, CH_2_-4), 3.7 (s, 3H, OCH_3_), 4.1 (s, 1H, CH-4, pyran), 6.2-6.9 (m, 7H, Ar), 7.1 (t, *J* = 7.6, 1H, C-4` of phenyl), 7.5 (d, *J* = 9.3, 2H, Ar), 7.6 (d, *J* = 9.3, 2H, Ar), 7.9-8.2 (m, 5H, Ar and NH_2_); ^13^C-NMR (DMSO-*d_6_*): *δ*, 22.2 (CH_2_-3), 26.5 (CH_2_-2), 29.8 (CH_2_-4), 33.1 (CH-4, pyran), 42.6 (C-5, pyran), 55.6 (OCH_3_), 66.8 (C-3, pyran), 107.0, 108.0, 110.8, 118.6, 119.8, 124.6, 125.2, 130.1, 130.7, 142.7, 143.0, 144.9, 146.3, 149.0, 153.8, 156.3, 157.2, 161.1, 161.5, 162.1, 165.9, 168.1, 169.0 (Ar-C and CN); MS: m/z (%): 600, 602 (M^+^, 18, 30) consistent with the molecular formula C_34_H_27_N_5_O_4_S. Anal. Calcd. C, 67.87; H, 4.52; N, 11.64; Found C, 67.56; H, 4.23; N, 11.89%.

#### 2.1.19. 2-((4-Bromophenyl)imino)-5-((dimethylamino)methylene)-3-((6-methoxy-3,4-dihydronaph thalene-1(*2H*)-ylidene)amino)thiazolidin-4-one 6

A mixture of compound **4a** (0.001 mol) and DMF-DMA (2 mL) was refluxed for ~1 h; after cooling, the solid product was filtered, washed with petroleum ether, dried, and recrystallized from dilute ethanol to give compound **6**. Yield: 87%; m.p.: 236°C; IR (*υ*_max_/cm^−1^): 1670 (C=O); ^1^H-NMR (DMSO-*d_6_*): *δ*; 1.7 (m, 2H, CH_2_-3), 2.59 (t, *J* = 6.1, 2H, CH_2_-2), 2.7 (t, *J* = 5.6, 2H, CH_2_-4), 3.6, 3.7 (2 s, 6H, -NMe_2_), 3.8, (s, 3H, OCH_3_), 6.7 (s, 1H, Ar, CH-5), 6.8 (d, *J* = 8.8, 1H, Ar, CH-7), 7.4 (d, *J* =8.6, 2H, Ar, CH-3`,5`), 7.5 (s, 1H, =CH-N), 7.7 (d, *J* =8.6, 2H, Ar, CH, 2`,6`) 8.04 (d, *J* = 8.8, 1H, Ar, CH-8); ^13^C-NMR (DMSO-*d_6_*): *δ*; 22.4 (CH_2_-3), 26.5 (CH_2_-2), 29.2 (CH_2_-4), 35.9 (-NMe_2_), 55.6 (OCH_3_), 86.2, 110.2, 110.6, 112.5, 113.6, 117.8, 124.8, 128.0, 128.1, 131.2, 139.1, 142.8, 149.6, 160.3, 167.4 (Ar-C, CN and C=O); MS: m/z (%): 498, 501 (M^+^, 58, 56), consistent with the molecular formula C_23_H_23_ Br N_4_O_2_S. Anal. Calcd. C, 55.31; H, 4.64; N, 11.22; Found C, 55.09; H, 4.54; N, 10.98%.

#### 2.1.20. Ethyl-2-((4-bromophenyl)imino)-3-((6-methoxy-3,4-dihydronaphthalen-1(*2H*)-ylidene) amino)-5-methyl-2,3-dihydrothiazolo [4,5-b]pyridine-6-carboxylate (7) and 2-((4-bromo phenyl)imino)-3-((6-methoxy-3,4-dihydronaphthalen-1(*2H*)-ylidene)amino)-5-methyl-2,3-dihydrothiazolo [4,5-b]pyridin-6-yl) ethanone 8

To a solution of compound **6** (0.005 mol) and ammonium acetated (0.5gm) in glacial acetic acid (5 mL), ethyl acetoacetoacetate or acetylacetone (0.005 mol) was added. The mixture was heated under reflux for 4 h.; after completion of the reaction and cooling, the product was poured onto ice cold water, dried, and recrystallized from the suitable solvent to give compounds **7** and **8**.

#### 2.1.21. Ethyl-2-((4-bromophenyl)imino)-3-((6-methoxy-3,4-dihydronaphthalen-1(*2H*)-ylidene) amino)-5-methyl-2,3-dihydrothiazolo [4,5-b]pyridine-6-carboxylate 7

Yield: 83%; recrystallized from ethyl alcohol; m.p.: 90°C; IR (*υ*_max_/cm^−1^): 1710 (C=O); ^1^H-NMR (DMSO-*d_6_*): *δ*, 1.2 (t, *J* = 6.8, 3H, -CH_2_-CH_3_), 1.7, (m, 2H, CH_2_-3), 2.1 (s, 3H, CH_3_, pyridine), 2.6 (t, *J =* 5.6, 2H, CH_2_-2), 2.7 (t, *J* = 5.3, 2H, CH_2_-4), 3.8 (s, 3H, OCH_3_), 4.1 (m, 2H, -CH_2_-CH_3_), 6.7 (s, 1H, Ar, H-5), 6.8 (d, *J* = 9.3, 1H, Ar, H-7), 7.3-7.7 (m, 5H, Ar), 8.0 (d, *J* = 9.3, 1H, Ar, CH-8); ^13^C-NMR (DMSO-*d_6_*): *δ*, 14.5 (CH_2_CH_3_), 18.4 (CH_3_- pyridine), 22.2 (CH_2_-3), 27.1 (CH_2_-2), 29.9 (CH_2_-4), 55.6 (OCH_3_), 61.01 (CH_2_CH_3_), 112.9, 113.0, 113.07, 115.5, 115.9, 123.1, 124.9, 125.4, 127.1, 130.7, 130.8, 132.3, 140.4, 148.6, 155.6, 162.5, 163.5, 170.3 (Ar-C, CN and C=O); MS: m/z (%): 564, 565.7, (M^+^, 54, 53) consistent with the molecular formula C_27_H_25_Br N_4_O_3_S. Anal. Calcd. C, 57.35; H, 4.46; N, 9.91; Found C, 57.10; H, 4.27; N, 9.86%.

#### 2.1.22. 2-((4-Bromophenyl)imino)-3-((6-methoxy-3,4-dihydronaphthalen-1(*2H*)-ylidene)amino)-5-methyl-2,3-dihydrothiazolo [4,5-b]pyridin-6-yl) ethanone 8

Yield: 80%; Crystallized from n-hexane; m.p.: 128°C; IR (*υ*_max_/cm^−1^): 1714 (C=O); ^1^H-NMR (DMSO-*d_6_*): *δ*, 1.7 (m, 2H, CH_2_-3), 2.1 (s, 3H, CH_3_, pyridine), 2.3 (s, 3H, COCH_3_), 2.7 (m, 2H, CH_2_-2), 2.9 (t, *J* = 5.9, 2H, CH_2_-4), 3.8 (s, 3H, OCH_3_), 6.7 (s, 1H, Ar, CH-5), 7.3-7.8 (m, 6H, Ar), 8.0 (d, *J* = 9, 1H, Ar, CH-8) ^13^C-NMR (DMSO-*d_6_*): *δ*, 18.2 (CH_3_, pyridine), 22.2 (CH_2_-3), 27.3 (CH_2_-2), 29.7 (CH_2_-4), 30.1 (COCH_3_), 55.7 (OCH_3_), 102.07, 103.1, 113.07,113.7, 121.9, 124.9, 126.9, 129.1, 130.7, 132.3, 134.9, 143.4, 147.6, 155.6, 161.1, 162.5, 163.5 (Ar-C and CN), 196.5 (C=O); MS: m/z (%): 535, 537 (M^+^, 37, 36) consistent with the molecular formula C_26_ H_23_ Br N_4_O_2_ S. Anal. Calcd. C, 58.32; H, 4.33; N, 10.46; Found C, 58.06; H, 3.97; N, 10.12%.

#### 2.1.23. 2-((4-Bromophenyl)imino)-3-((6-methoxy-3,4-dihydronaphthalen-1(*2H*)-ylidene)amino)-5-(oxo/thioxo)-2,3,4,5-tetrahydrothiazolo [4,5-b]pyridine-6-carbonitrile 9 and 10

To a solution of compound **6** (0.005 mol) in ethanolic sodium hydroxide solution (0.12gm sodium metal in 20 mL absolute ethanol), malononitrile or cyanothioacetamide (0.005 mol) was added. The mixture was refluxed for 3 h, the excess solvent was evaporated under reduced pressure, and the solid product was collected by filtration, washed with water, dried, and recrystallized from ethanol to give the products **9** and **10**, respectively.

#### 2.1.24. 2-((4-Bromophenyl)imino)-3-((6-methoxy-3,4-dihydronaphthalen-1(*2H*)-ylidene)amino)-5-oxo-2,3,4,5-tetrahydrothiazolo [4,5-b]pyridine-6-carbonitrile 9

Yield: 83%; m.p.: 224°C; IR (*υ*_max_/cm^−1^): 3330 (NH), 2212 (CN), 1660 (C=O); ^1^H-NMR (DMSO-*d_6_*): *δ*, 1.7 (m, 2H, CH_2_-3), 2.6 (t, *J* = 6.2, 2H, CH_2_-2), 2.73 (t, *J* = 5.7, 2H, CH_2_-4), 3.7 (s, 3H, OCH_3_), 6.7 (s, 1H, Ar, CH-5), 6.8 (d, *J* = 8.8, 1H, Ar, H-7), 7.3 (m, 2H, Ar,), 7.5 (s, 1H, CH-4, pyridine), 7.6 (m, 2H, Ar), 8.0 (d, *J* = 8.8, 1H, Ar, CH-8), 8.5 (s, 1H, NH). ^13^C-NMR (DMSO-*d_6_*): *δ*, 22.2 (CH_2_-3), 27.2 (CH_2_-2), 29.9 (CH_2_-4), 55.6 (OCH_3_), 87.8 (C-5b pyridine), 112.9, 113.7, 116.2, 121.1, 125.4, 126.8, 130.7, 131.7, 132.1, 135.4, 142.8, 144.8, 159.0, 159.02, 160.8, 166.9, 167.1 (Ar-C, CN and C=O); MS: m/z (%):518, 520 (M^+^, 45, 44) consistent with the molecular formula C_24_H_18_BrN_5_O_2_S. Anal. Calcd. C, 55.39; H, 3.49; N, 13.46; Found C, 55.09; H, 3.12; N, 13.18%.

#### 2.1.25. 2-((4-Bromophenyl)imino)-3-((6-methoxy-3,4-dihydronaphthalen-1(*2H*)-ylidene)amino)-5-thioxo -2,3,4,5-tetrahydrothiazolo [4,5-b]pyridine-6-carbonitrile 10

Yield: 81%; m.p.: 286°C; IR (*υ*_max_/cm^−1^): 3380 (NH), 2220 (CN); ^1^H-NMR (DMSO-*d_6_*): *δ*, 1.7 (m, 2H, CH_2_-3), 2.58 (t, *J* = 6.3, 2H, CH_2_-2), 2.7 (t, *J* = 5.4, 2H, CH_2_-4), 3.77 (s, 3H, OCH_3_), 6.7 (s, 1H, Ar, CH-5), 6.8 (d, *J* = 8.7, 1H, Ar, CH-7), 7.4 (d, *J* =8.6, 2H, Ar), 7.5 (s, 1H, pyridine), 7.6 (d, *J* =8.6, 2H, Ar), 8.0 (d, *J* = 8.7, 1H, Ar, CH-8), 8.5 (s, 1H, NH); ^13^C-NMR (DMSO-*d_6_*): *δ*, 22.2 (CH_2_-3), 27.2 (CH_2_-2), 29.9 (CH_2_-4), 55.6 (OCH_3_), 85.3 (C-5b pyridine), 112.9, 113.7, 116.2, 121.1, 125.4, 126.8, 130.7, 131.7, 132.0, 135.4, 142.7, 144.8, 159.0, 159.9, 160.8, 166.8, 167.1 (Ar-C, CN and C=S); MS: m/z (%): 535,537 (M^+^, 26, 25,) consistent with the molecular formula C_24_H_18_BrN_5_OS_2._ Anal. Calcd. C, 53.73; H, 3.38; N, 13.05; Found C, 53.25; H, 3.13; N, 12.86%.

#### 2.1.26. 5-((1H-Benzo [d]imidazol-2-ylamino)methylene)-2-(4-bromophenylimino)-3-((6-methoxy-3,4 -dihydronaphthalen-1(*2H*)-ylidene)amino)thiazolidin-4-one 11 and 2-(4-bromophenyl-imino)-3-((6-methoxy-3,4-dihydronaphthalen-1(*2H*)-ylidene)amino)-5-((thiazol-2-ylamino) methylene)thiazolidin-4-one 12

To a mixture of the enaminone **6** (0.005 mol) in glacial acetic acid (15 mL), 2-aminobenz- imidazole or 2-aminothiazole was added. The mixture was allowed to react under reflux for 2 h., and the excess solvent was evaporated under vacuum. The solid was collected by filtration, washed with water, dried, and recrystallized from the suitable solvent to give the products **11**and **12**.

#### 2.1.27. 5-((1H-Benzo [d]imidazol-2-ylamino)methylene)-2-(4-bromophenylimino)-3-((6-methoxy-3,4 -dihydronaphthalen-1(*2H*)-ylidene)amino)thiazolidin-4-one 11

Yield: 78%; crystallized from ethyl alcohol; m.p.: 220°C; IR (*υ*_max_/cm^−1^): 3368, 3290 (2NH), 1665 (C=O); ^1^H-NMR (DMSO-*d_6_*): *δ*, 2.09 (m, 2H, CH_2_-3), 2.7 (t, *J* = 6.3, 2H, CH_2_-2), 2.9 (t, *J* = 5.7, 2H, CH_2_-4), 3.7 (s, 3H, OCH_3_), 3.8 (s, 1H, NH), 6.6-6.9 (m, 3H, Ar), 7.3-8.0 (m, 9H, Ar,), 8.4 (s,1H, NH); ^13^C-NMR (DMSO-*d_6_*): *δ*, 22.0 (CH_2_-3), 23.3 (CH_2_-2), 29.7 (CH_2_-4), 55.6 (OCH_3_), 110.9, 112.9, 113.04, 113.7, 116.4 124.1, 126.9, 129.2, 130.6, 132.3, 133.5, 133.8, 133.8, 142.5, 148.2, 161.5, 161.7, 162.4, 163.6, 174.2.0 (Ar-C, CN and C=O); MS: m/z (%):586,588 (M^+^, 100, 98) consistent with the molecular formula C_28_H_23_BrN_6_O_2_S. Anal. Calcd. C, 57.24; H, 3.95; N, 14.31; Found C, 56.97; H, 3.74; N, 14.02%.

#### 2.1.28. 2-(4-Bromophenylimino)-3-((6-methoxy-3,4-dihydronaphthalen-1(*2H*)-ylidene)amino)-5-((thiazol-2-ylamino)methylene)thiazolidin-4-one 12

Yield: 79%; recrystallized from isopropyl alcohol; m.p.: 152°C; IR (*υ*_max_/cm^−1^): 3380 (NH), 1660 (C=O); ^1^H-NMR (DMSO-*d_6_*): *δ*, 2.08 (m, 2H, CH_2_-3), 2.4 (t, *J* = 6.3, 2H, CH_2_-2), 2.7 (t, *J* = 5.7, 2H, CH_2_-4), 3.5 (s, 1H, NH), 3.7 (s, 3H, OCH_3_), 6.7 (d, *J* = 8.5, 1H, Ar), 6.8 (d, 1H, *J* =8.6, Ar), 7.1-7.4 (m, 7H, Ar,), 7.9 (d, *J* = 8.9, 1H, Ar); ^13^C-NMR (DMSO-*d_6_*): *δ*, 22.0 (CH_2_-3), 23.3 (CH_2_-2), 29.0 (CH_2_-4), 55.6 (OCH_3_), 109, 112.9, 113.04, 113.7, 124.1, 126.1, 126.9, 129.2, 130.6, 132.3, 133.5, 133.8, 139.8, 148.2, 161.5, 161.7, 163.6, 170.1 (Ar-C and CN); MS: m/z (%): 553, 555 (M^+^, 50, 49) consistent with the molecular formula C_24_H_20_BrN_5_O_2_S_2_, Anal. Calcd. C, 51.99; H, 3.64; N, 12.63; Found C, 51.78; H, 3.46; N, 12.49%.

### 2.2. Cell Lines and Cell Culture

Both MCF-7 and MCF10A cells were purchased from American Type Culture Collection (ATCC). Cells were grown in DMEM culture medium (Invitrogen/Life Technologies) supplemented with 10% FBS (Hyclone, USA), 10 *μ*g/mL insulin (Sigma), and 1% penicillin-streptomycin antibiotic solution. Chemicals used were of cell culture grade and were purchased from Sigma or Invitrogen. Prior to the assay, cells (cells density 1.2–1.8 × 10,000 cells/well) were plated in 96-well plate with 100 *μ*L medium and were allowed to grow for 24 h.

### 2.3. *In Vitro* Cytotoxicity Assay

*In vitro* cytotoxic activity of the prepared compounds against breast (MCF-7) cancer cells was assessed using MTT assay [24, 25]. The assay depends on the mitochondrial reduction of the colorless 3-(4,5-methyl-2-thiazolyl)-2,5-diphenyl-2H-tetrazolium bromide (MTT) within viable cells into a dark blue formazan product. Cells were cultured in DMEM medium supplemented with 10% FBS at a final concentration of 2 × 10^4^ cells/mL in 96-well plates and incubated in a 5% CO_2_ incubator at 37°C. Twelve hours later, different concentrations (0.39-100 *μ*M) of the tested compound (2 *μ*L) were added to the cells (2 × 10^4^) in 96-well plates and cultured at 37°C for 3 days. Then, 20 *μ*L of MTT solution was added to the cultured cells and incubated for four hours at 37°C. The supernatant was taken away from each well, and 100 *μ*L of DMSO was added to each well to dissolve the formazan crystals. After mixing with a mechanical plate mixer, a microplate reader was used to measure the absorbance of each well at a wavelength of 570 nm. Data were expressed as IC_50_ (*μ*M), i.e., the concentration required to inhibit 50% of viable cell growth. IC_50_ values were calculated from the linear regression of the corresponding calibration curves using the Origin® 6.1 software. Each experiment was carried out in triplicate with good reproducibility and standard errors.

### 2.4. Statistical Analysis

Results were analyzed with the help of SPSS 9.0 and were presented as mean ± SD of three replicates. The mean comparison between different evaluated groups was performed using ANOVA one-way analysis of variance. Statistical significance was defined when *p* < 0.05.

## 3. Results and Discussion

### 3.1. Chemistry

**Scheme 1:**
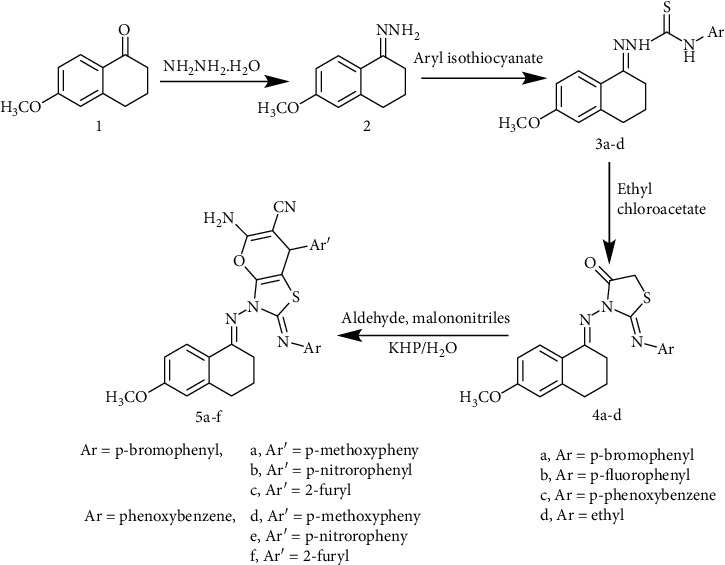
Synthetic route of compounds 2-5a-f.

Starting with 6-methoxy-1-tetralone **1**, its hydrazine derivative **2**, was prepared as previously reported method [23], reaction of the hydrazine derivative **2** with different aryl isothiocyanates, namely, p-bromophanyl, p-flourophanyl, p-phenoxybenzene, and ethyl isothiocyanates produced the desired thiosemicarbazides **3a-d** in 85-94% yield. Thiosemicarbazides **3a-d** was separately refluxed with ethylchloroacetate in ethanol to give the thiazolidinone derivatives **4a-d** in 76-94% yields. Pyrano thiazole-carbonitrile derivatives **5a-f** were prepared in 55-81% yields, by heating a mixture of compound **4a** or **4c**, different aryl aldehydes namely, p-methoxy benzaldehyde, p-nitrobenzaldehyde, and/or 2-furaldehyde at 50°C and malononitrile utilizing distilled water as a solvent and in the presence of catalytic amount of potassium hydrogen phthalate (Scheme 1).

**Scheme 2:**
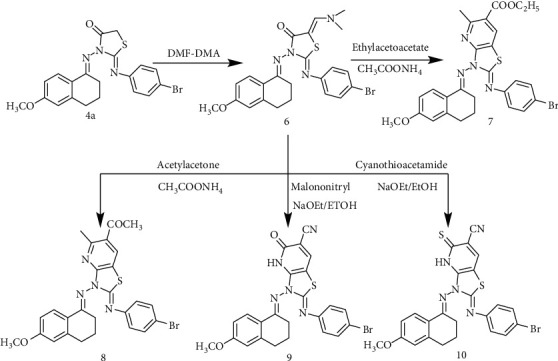
Synthetic route of compounds 6-10.

On the other hand, synthesis of the enaminone, **6** was essential to construct biologically active heterocyclic products. Reaction of the thiazolidinone **4a** with dimethylformamide-dimethylacetal (DMF-DMA), under solvent-free conditions, produced the enaminone derivative **6** within 1 h., in 87% yield. The enamine **6** was condensed with ethyl acetoacetate or acetyl acetone in the presence of ammonium acetate and glacial acetic acid, to afford the thiazolopyridine carboxalate and thiazolo-pyridine ethanone derivatives **7**and **8**, respectively, in 83 and 80% yields. While, reaction of compound **6** with cyanothioacetamide or malononitrile in ethanolic sodium ethoxide gave thioxo-pyridine or oxo-pyridine carbonitrile derivatives **9** and **10**, respectively, in 83 and 81% yields (Scheme 2**).**

**Scheme 3:**
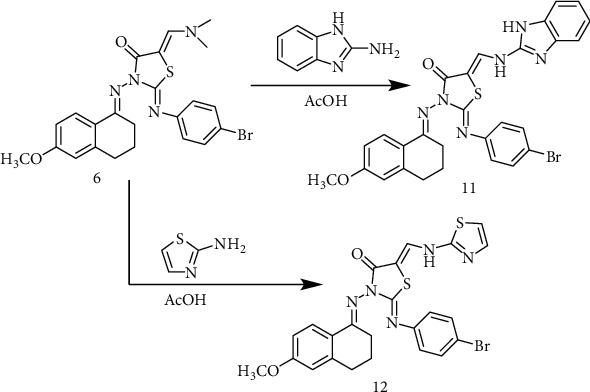
Synthetic route of compounds 11 and 12.

Finally, reaction of the enaminone **6** with 2-aminoimidazol or 2-aminothiazol in the presence of glacial acetic acid produced imidazol-thiazolidinone and thiazolo-thiazolidinone derivatives **11** and **12** in 78 and 79% yields, respectively (Scheme 3).

### 3.2. Cytotoxic Screening

**Figure 1:**
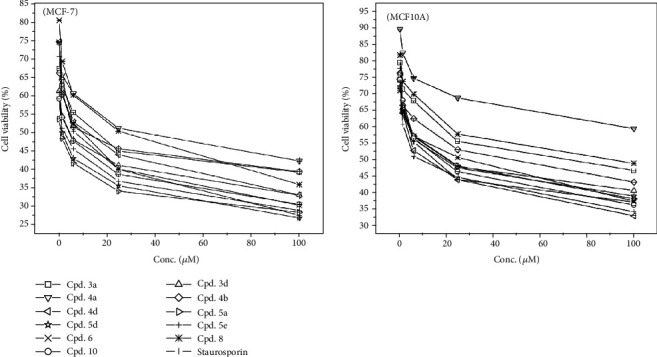
Effect of different concentrations of each prepared compound on cell viability of breast cancer cell lines (MCF-7) and normal breast cells (MCF10A).

Eleven compounds were investigated *in vitro* for their activities against breast cancer cell line MCF-7 using MTT assay. The effect of different concentrations of the newly synthesized compounds was evaluated by determining the percentages of viable cells after being exposed to the applied concentrations, compared to Staurosporin as a reference drug. In this screening, all the tested compounds showed potential cytotoxic activities against MCF-7 cells in a dose-dependent manner (Figure 1). Furthermore, it can be seen that the compounds affected cell viability in different patterns. This can be attributed to the differences of cellular response to each compound, depending on the nature of its terminal and functional groups [26, 27]. Results demonstrated that five of the tested compounds **5a**, **5d**, **5e**, **10**, and **3d** showed potential cytotoxic activities against breast cancer cell line MCF-7, recording IC_50_ values of 0.93 ± 0.02, 1.76 ± 0.04, 2.36 ± 0.06, 2.83 ± 0.07, and 3.73 ± 0.09 *μ*M, respectively (Table 1). Additionally, it can be seen that these potential compounds had cytotoxic activities higher than the tested positive control (Saturosporin, IC_50_6.08 ± 0.15 *μ*M). Comparing these results with those obtained against normal breast cell line (MCF10A) showed that the tested compounds were less toxic toward normal cells. Compound **5a** was the most effective against MCF-7 cells and was less toxic than Saturosporin by about 18.45-folds towards MCF01A normal cells. The acquired data revealed that coupling the pyrano ring in this fused heterocyclic ring system was critically influenced the cytotoxic activity. All the tested pyrane containing compounds (**5a**, **5d**, and **5e**) were the most effective cytotoxic agents and were more potent than Staurosporin (IC_50_; 0.93, 1.76, and 2.36 *μ*M, respectively, IC_50_ Staurosporin; 6.08 *μ*M). The size of N-substitution in thiourea-imino-linked to hydronaphthaline core directly affected the cytotoxic activity. Accordingly, the N-ethyl thiourea derivative **3d** had a very good cytotoxicity (IC_50_; 3.73 *μ*M). On the other hand, the N-4-bromophenyl substitution of thiourea in analog **3a** resulted in more than 3 times decrease in cytotoxicity. Also, a closer antiproliferative potency was exhibited in compound **10** (IC_50_; 2.83 *μ*M), in which our core was imino-linked to 5-thioxo-2,3,4,5-tetrahydrothiazole [4,5-b]pyridine-6-carbonitrile ring system. While, the methylation of C-5 and acetylation of C-6 of the dihydrothiazolo [4,5-b] pyridinyl ring system in compound **8** yielded a significantly weaker cytotoxic activity than compound **10** (IC_50_; 20 *μ*M). This highlighted the importance of 5-thioxo and 6-carbonitrile functionality for MCF7 antiproliferative activity in this fused heterocyclic ring system.

## 4. Conclusion

In the course of our research work, some new dihydronaphthalene derivatives were synthesized starting with 6-methoxy-1-tetralone **1**. Cytotoxic evaluation of eleven compounds was estimated against MCF-7 human cancer cells (breast cancer) utilizing Staurosporin as a reference drug. Results declared that compounds **5a**, **5d**, **5e**, **10,** and **3d** appeared to be the most active products of IC_50_ values; 0.93 ± 0.02, 1.76 ± 0.04, 2.36 ± 0.06, 2.83 ± 0.07, and 3.73 ± 0.09 *μ*M, respectively, which were more potent than the reference used (Saturosporin, IC_50_6.08 ± 0.15 *μ*M). These active products possessed selectivity and showed lower toxicity than the reference drug. Compound **5a** was the most effective against MCF-7 cells and was less toxic than Saturosporin by about 18.45-folds towards MCF01A normal cells. At the same time, the tested products possessed selectivity and showed lower toxicity than the standard drug used when examined towards the breast normal cells.

## Figures and Tables

**Table 1:** IC_50_ values of the prepared compounds against breast cancer (MCF-7) and breast normal (MCF10A) cell lines.

Comp. No.	IC_50_ (*μ*M)	
	MCF-7	MCF10A
3a	12.66 ± 0.33	35.42 ± 0.64
3d	3.73 ± 0.09	19.73 ± 0.51
4a	28.62 ± 0.74	67.95 ± 0.86
4b	10.22 ± 0.26	29.15 ± 0.36
4d	10.11 ± 0.26	30.74 ± 0.53
5a	0.93 ± 0.02	17.16 ± 0.44
5d	1.76 ± 0.04	16.33 ± 0.42
5e	2.36 ± 0.06	14.06 ± 0.36
6	7.48 ± 0.19	17.78 ± 0.16
8	20.01 ± 0.52	43.69 ± 0.59
10	2.83 ± 0.07	15.01 ± 0.39
Staurosporin	6.08 ± 0.15	9.35 ± 0.24

## Data Availability

All data generated in this current work are included in the “Results and Discussion” section.

## Supplementary Materials

Supplementary Materials The supplementary file includes the Spectroscopic charts which support the study.
