# Supplementary material for: Synthesis and Characterization of New Dihydronaphthalene Candidates as Potent Cytotoxic Agents against MCF-7 Human Cancer Cells
Source: Biomed Res Int. 2020 Dec 23;2020:8649745. doi: 10.1155/2020/8649745 (PMC7787731; doi:10.1155/2020/8649745)

Microanalytical Unit - FOPCU - NMR laboratory
www.pharma.cu.edu.eg dir-mau.fopcu@pharma.cu.edu.eg

BRUKER

```
Current Data Parameters
NAME        Nesreen Saied_H_2
EXPNO                      10
PROCNO                      1

F2 - Acquisition Parameters
Date_                20191002
Time                    14.54
INSTRUM                 spect
PROBHD    5 mm PABBO BB/
PULPROG                  zg30
TD                      65536
SOLVENT                  DMSO
NS                         32
DS                          2
SWH               8012.820 Hz
FIDRES            0.122266 Hz
AQ               4.0894465 sec
RG                      180.8
DW                 62.400 usec
DE                   6.50 usec
TE                    298.0 K
D1             1.00000000 sec
TD0                         1

======== CHANNEL f1 ========
SFO1          400.1924713 MHz
NUC1                       1H
P1                  15.00 usec
PLW1            10.39999962 W

F2 - Processing parameters
SI                      65536
SF            400.1900000 MHz
WDW                        EM
SSB      0
LB                    0.30 Hz
GB       0
PC                       1.00
```

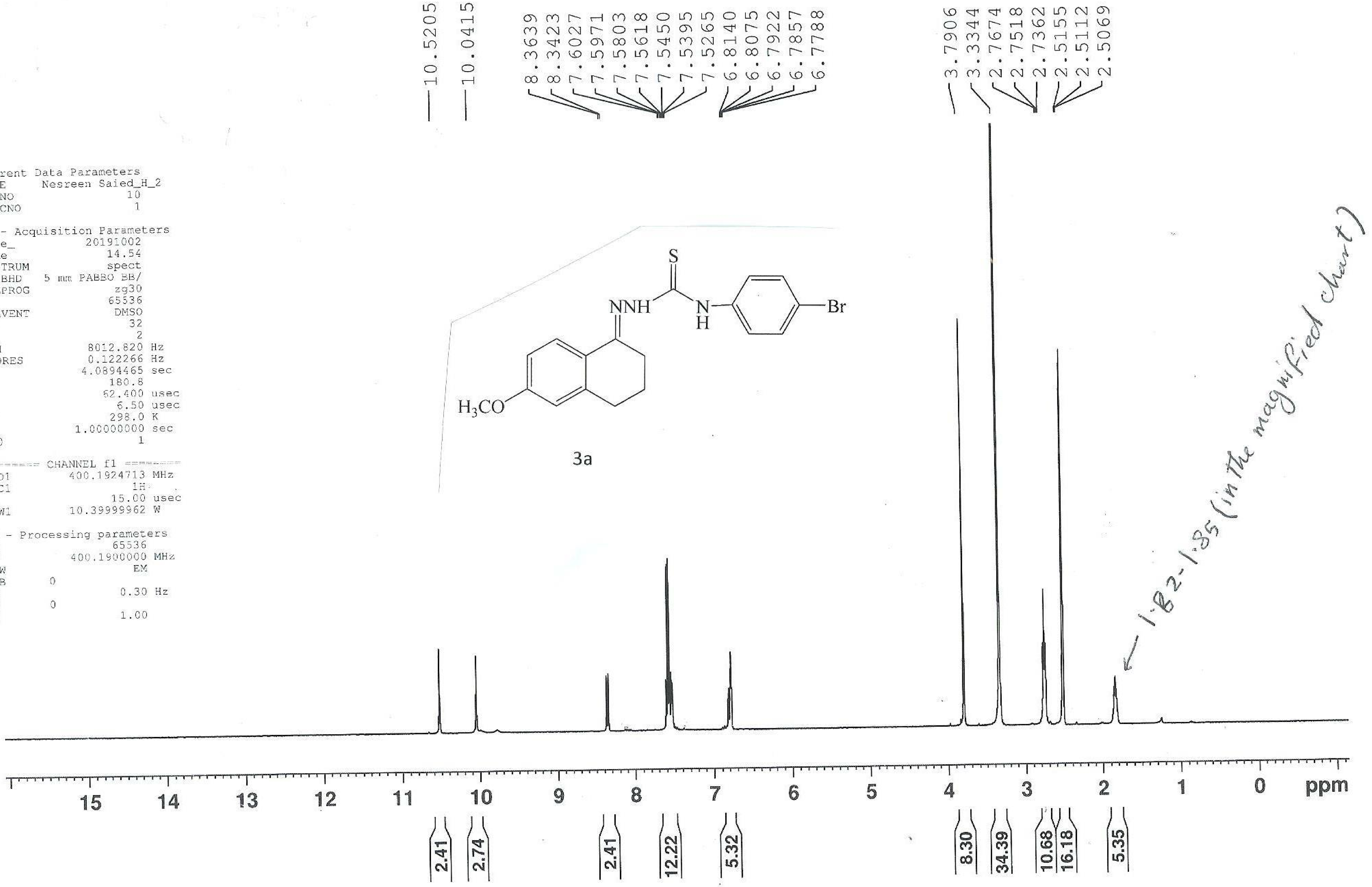

3a

2

Microanalytical Unit - FOPCU - NMR laboratory
www.pharma.cu.edu.eg dir-mau.fopcu@pharma.cu.edu.eg

BRUKER

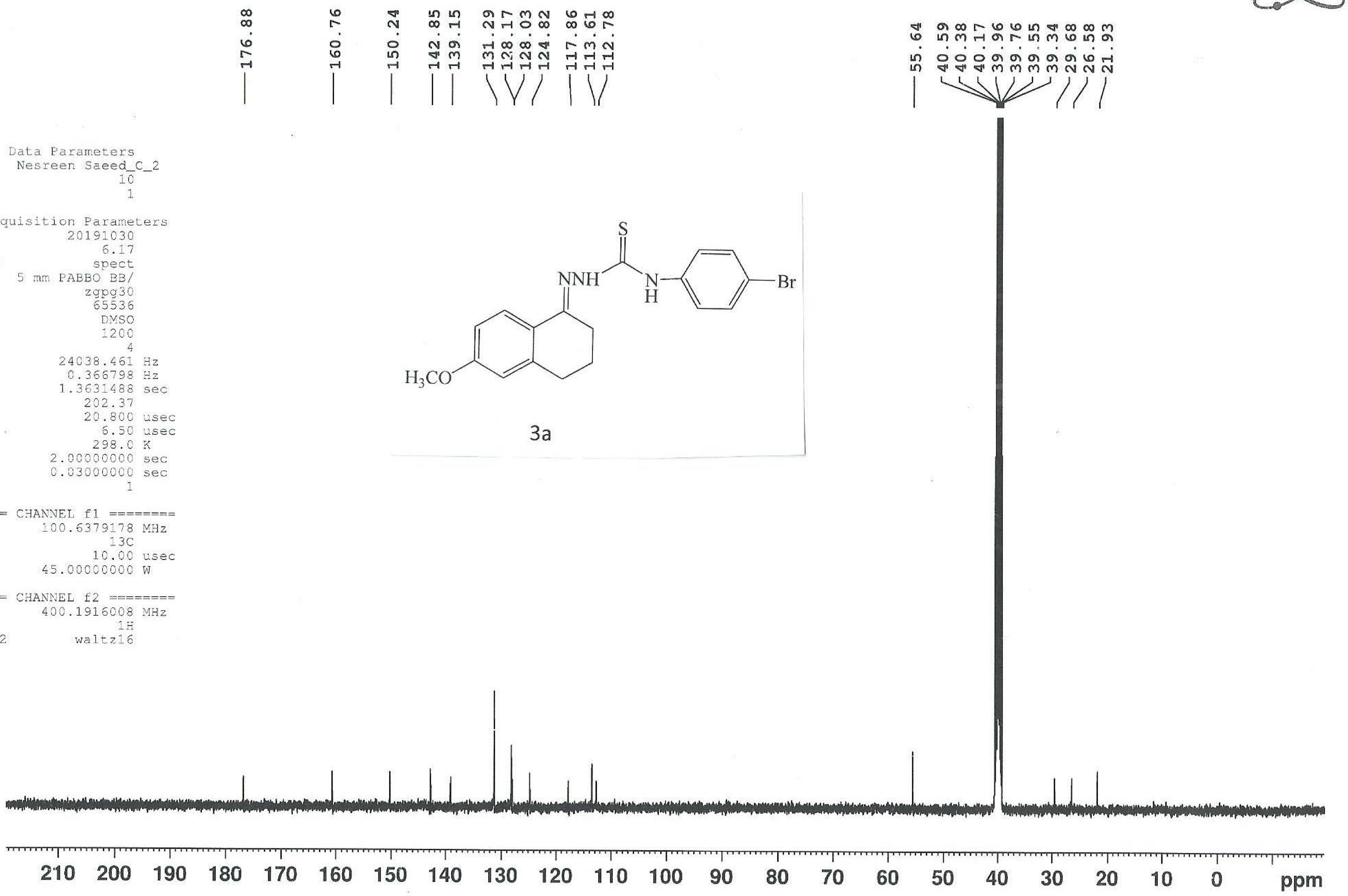

Current Data Parameters
NAME Nesreen Saeed_C_2
EXPNO 10
PROCNO 1

F2 - Acquisition Parameters
Date_ 20191030
Time 6.17
INSTRUM spect
PROBHD 5 mm PABBO BB/
PULPROG zgpg30
TD 65536
SOLVENT DMSO
NS 1200
DS 4
SWH 24038.461 Hz
FIDRES 0.366798 Hz
AQ 1.3631488 sec
RG 202.37
DW 20.800 usec
DE 6.50 usec
TE 298.0 K
D1 2.00000000 sec
D11 0.03000000 sec
TD0 1

======== CHANNEL f1 ========
SFO1 100.6379178 MHz
NUC1 13C
P1 10.00 usec
PLW1 45.00000000 W

======== CHANNEL f2 ========
SFO2 400.1916008 MHz
NUC2 1H
CPDPRG[2 waltz16

3a

C:\Xcalibur\...\Ali\EI-MS\2019\11\Alaa-2 05/11/2019 8:30:47 AM

Alaa-2 #33 RT: 0.14 AV: 1 NL: 2.36E4
T: {0,0} + c EI Full ms [50.00-500.00]

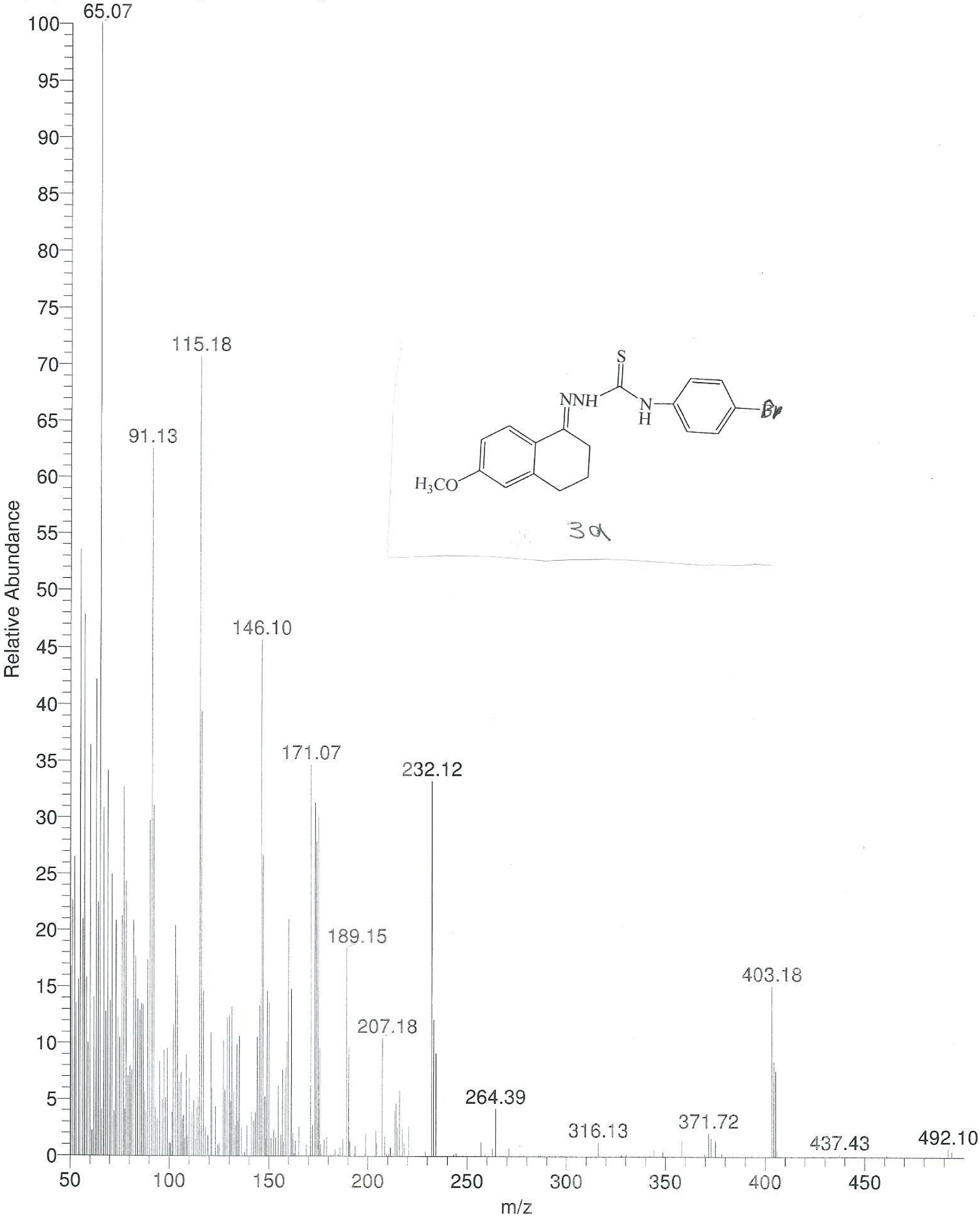

3a

(4)

Microanalytical Unit - FOPCU - NMR laboratory
www.pharma.cu.edu.eg dir-mau.fopcu@pharma.cu.edu.eg

BRUKER

```
Current Data Parameters
NAME      Nesreen Saied_H_3
EXPNO                   10
PROCNO                   1

F2 - Acquisition Parameters
Date_             20191002
Time                 15.04
INSTRUM              spect
PROBHD   5 mm PABBO BB/
PULPROG               zg30
TD                   65536
SOLVENT               DMSO
NS                      32
DS                       2
SWH            8012.820 Hz
FIDRES         0.122266 Hz
AO            4.0894465 sec
RG                   32.12
DW              62.400 usec
DE                6.50 usec
TE               298.0 K
D1          1.00000000 sec
TD0                      1

======== CHANNEL f1 ========
SFO1        400.1924713 MHz
NUC1                    1H
P1               15.00 usec
PLW1       10.39999962 W

F2 - Processing parameters
SI                   65536
SF          400.1900000 MHz
WDW                     EM
SSB      0
LB                 0.30 Hz
GB       0
PC                    1.00
```

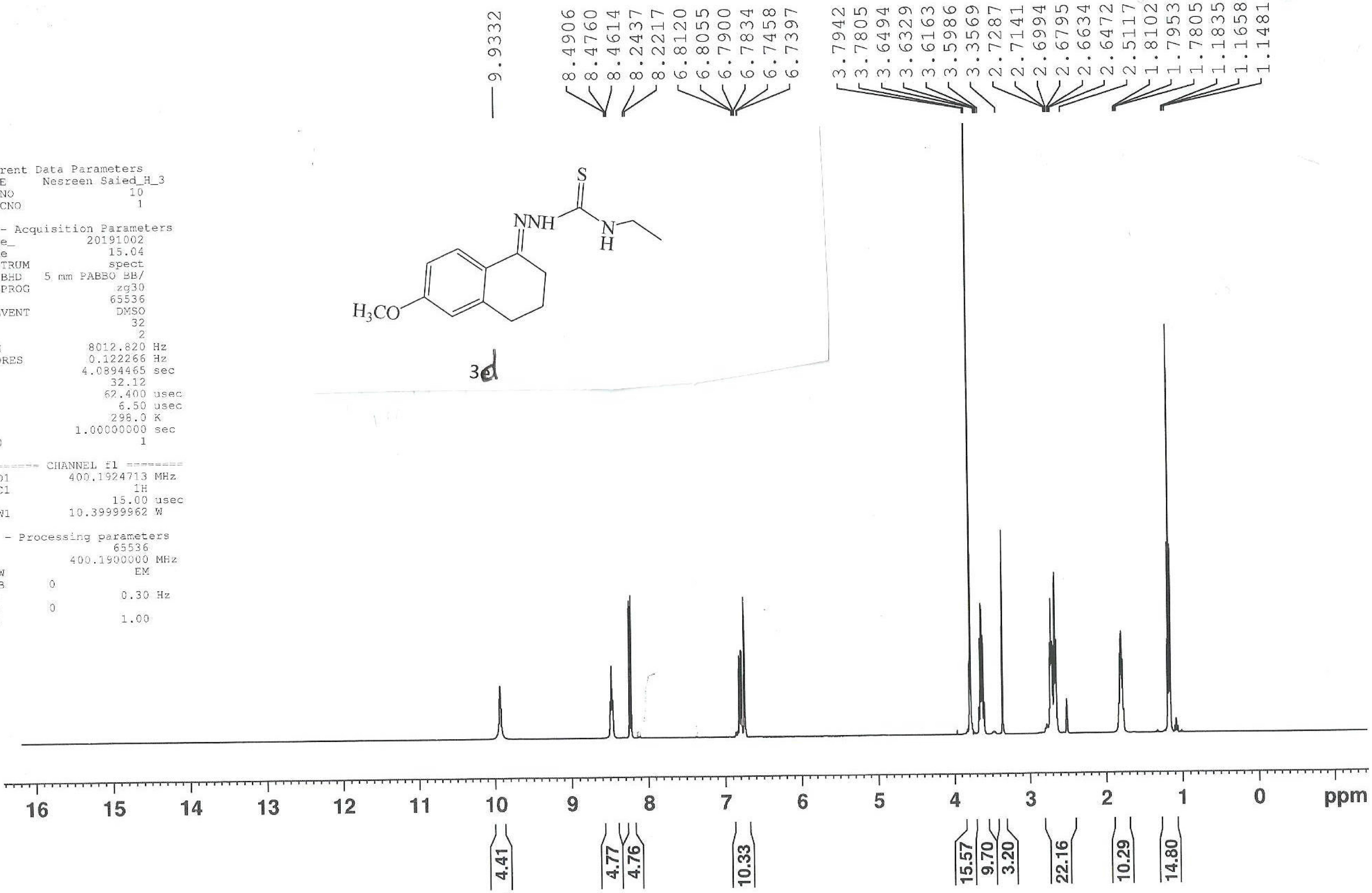

Microanalytical Unit - FOPCU - NMR laboratory
www.pharma.cu.edu.eg dir-mau.fopcu@pharma.cu.edu.eg

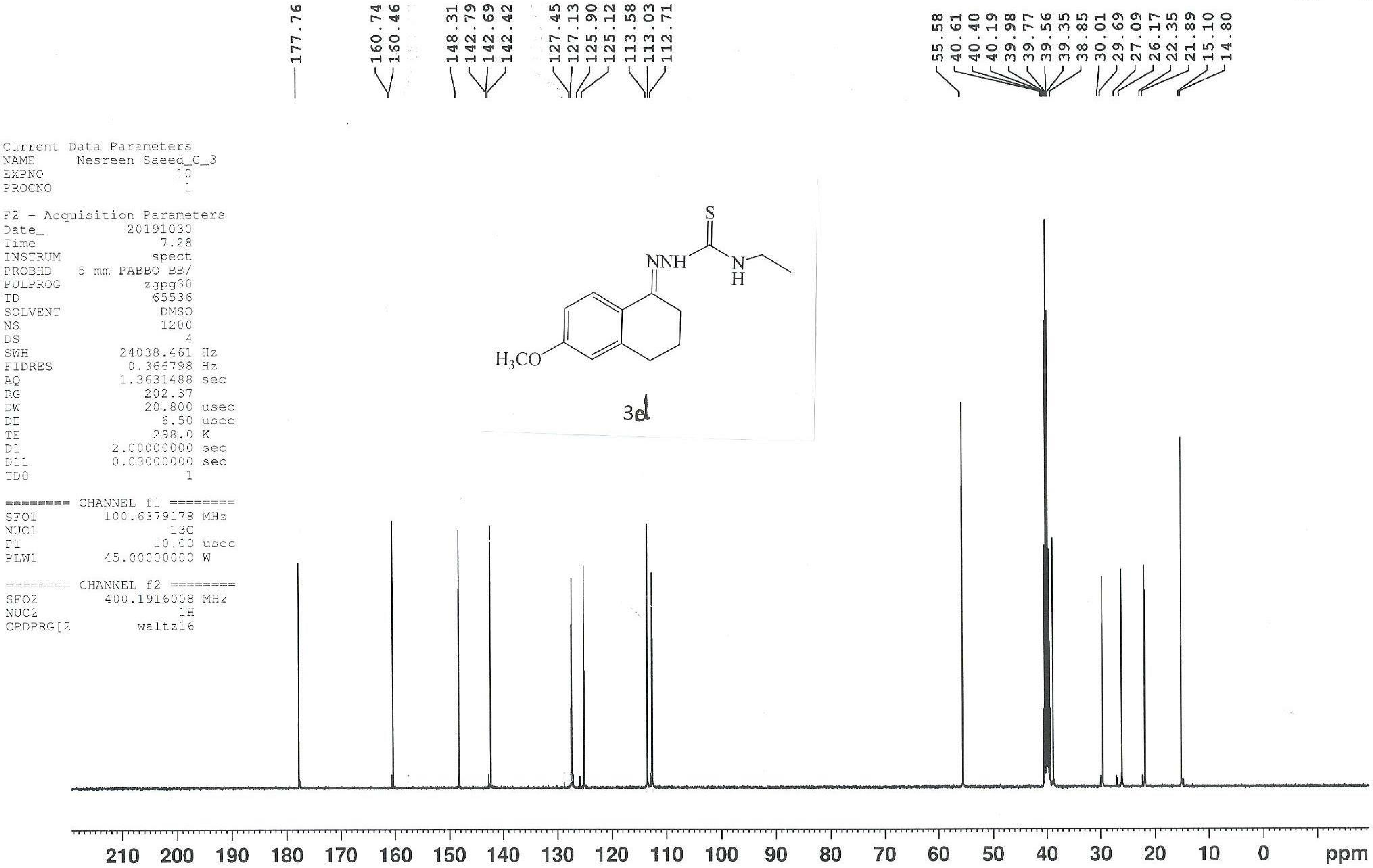

C:\Xcalibur\...\Ali\EI-MS\2019\11\Alaa-4 05/11/2019 8:37:07 AM

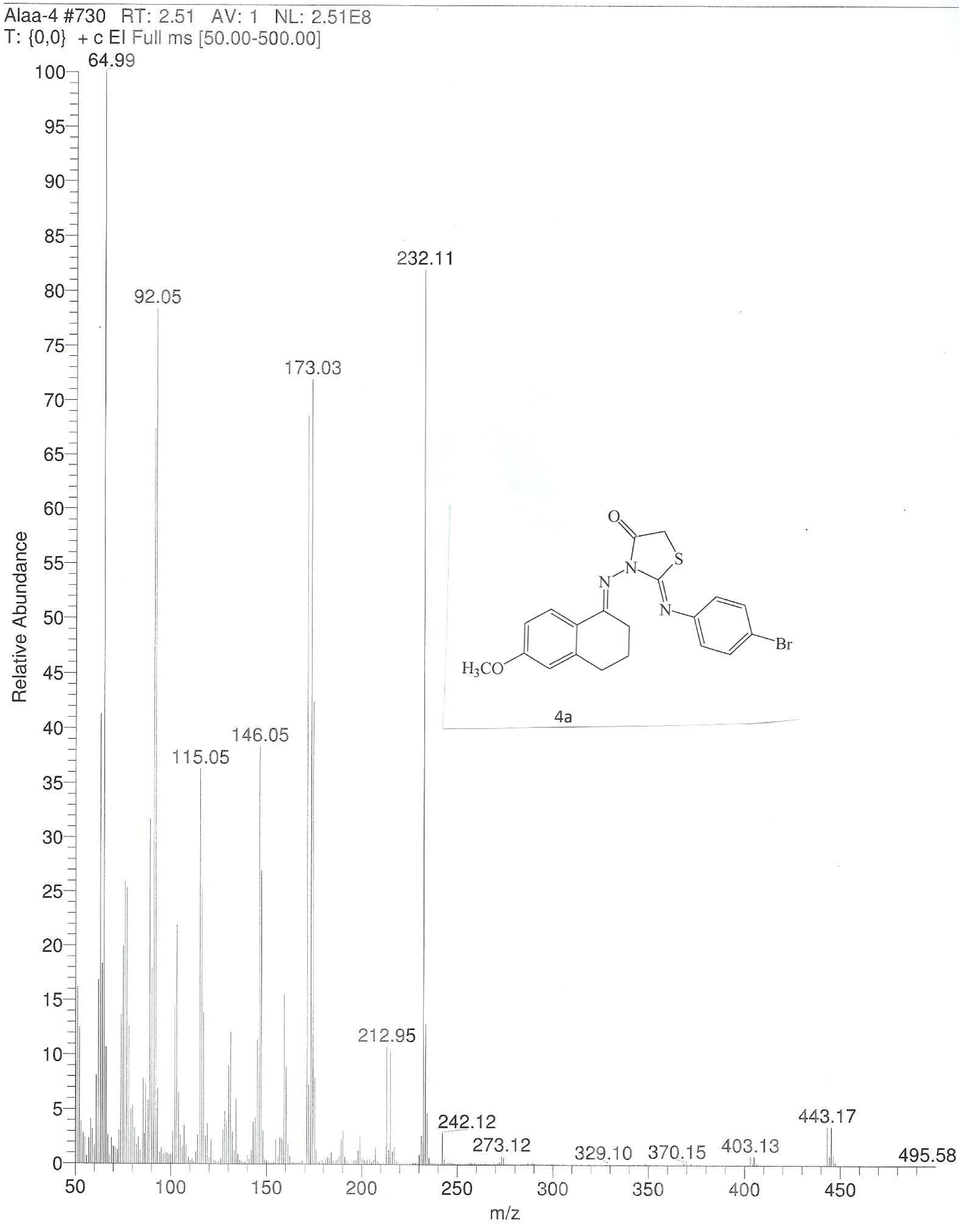

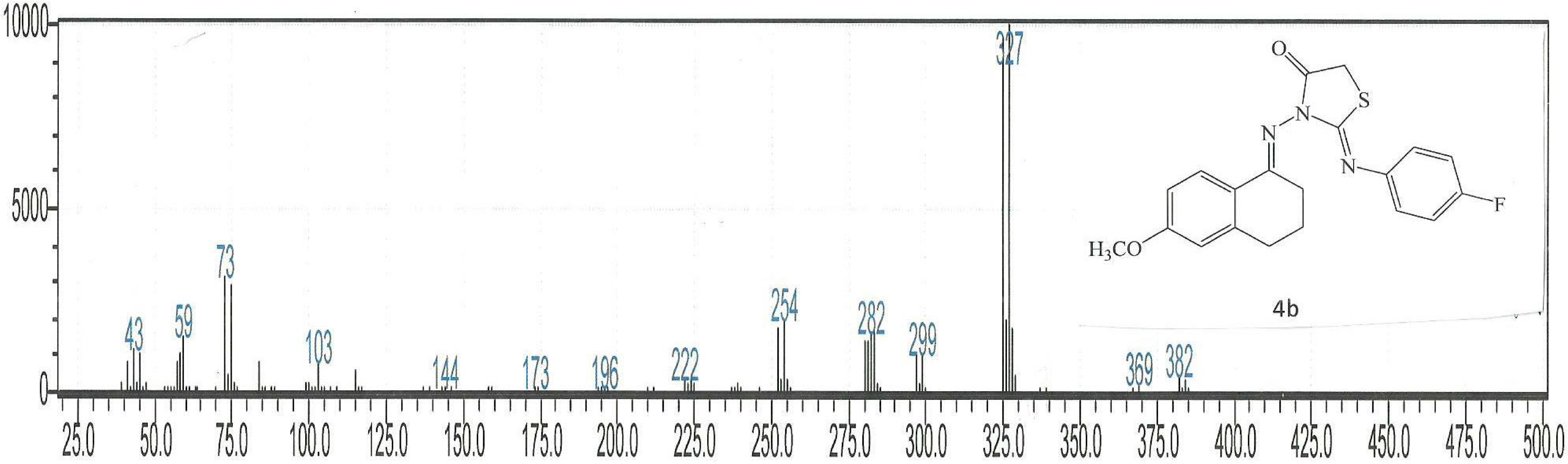

10000
5000
0
327
73
43
59
103
144
173
196
222
254
282
299
369
382
25.0
50.0
75.0
100.0
125.0
150.0
175.0
200.0
225.0
250.0
275.0
300.0
325.0
350.0
375.0
400.0
425.0
450.0
475.0
500.0
O
S
N
N
N
F
H3CO
4b

Microanalytical Unit - FOPCU - NMR laboratory
www.pharma.cu.edu.eg dir-mau.fopcu@pharma.cu.edu.eg

BRUKER

```
Current Data Parameters
NAME        Nesreen Saieed_H_5
EXPNO                       10
PROCNO                       1

F2 - Acquisition Parameters
Date_               20190806
Time                   16.25
INSTRUM                spect
PROBHD     5 mm PABBO BB/
PULPROG                 zg30
TD                     65536
SOLVENT                 DMSO
NS                        32
DS                         2
SWH                 8012.820 Hz
FIDRES              0.122266 Hz
AQ                 4.0894465 sec
RG                    169.46
DW                    62.400 usec
DE                      6.50 usec
TE                     298.0 K
D1                1.00000000 sec
TD0                        1

======== CHANNEL f1 ========
SFO1            400.1924713 MHz
NUC1                     1H
P1                     15.00 usec
PLW1             10.39999962 W

F2 - Processing parameters
SI                     65536
SF               400.1900000 MHz
WDW                       EM
SSB       0
LB                      0.30 Hz
GB        0
PC                      1.00
```

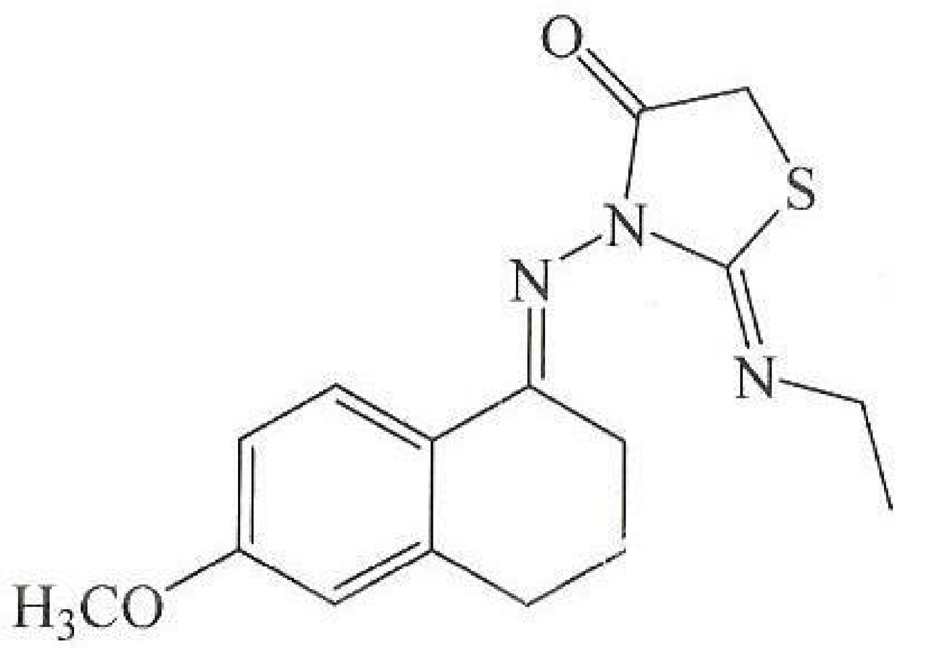

4d

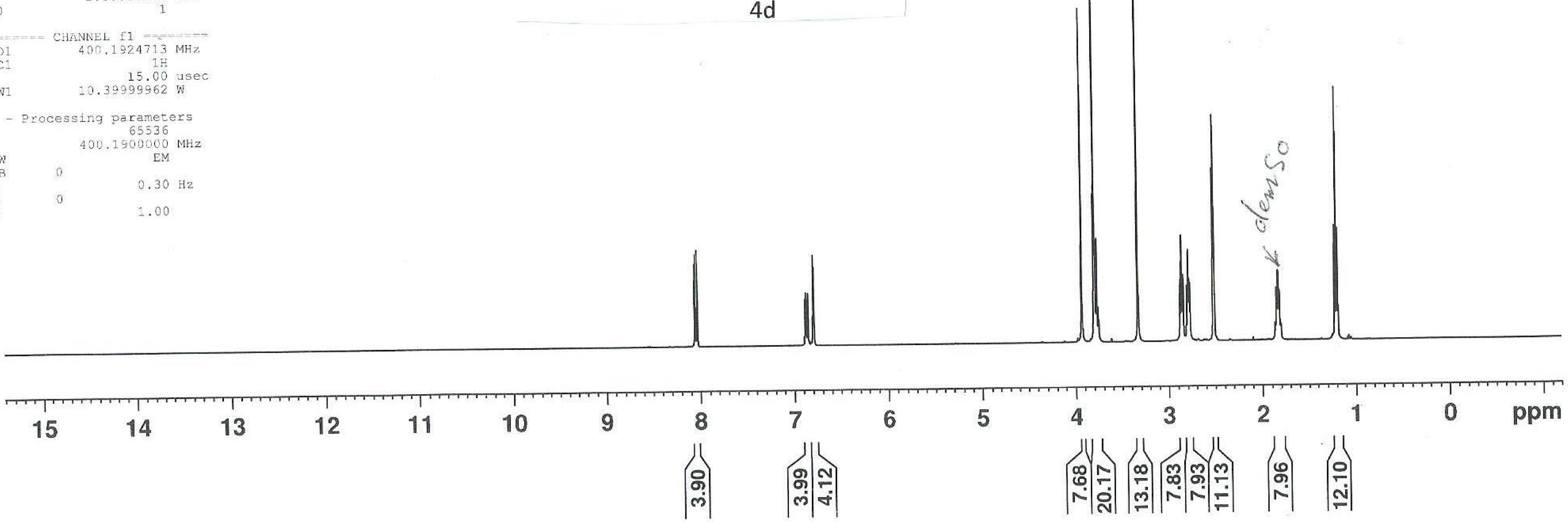

Microanalytical Unit - FOPCU - NMR laboratory
www.pharma.cu.edu.eg dir-mau.fopcu@pharma.cu.edu.eg

BRUKER

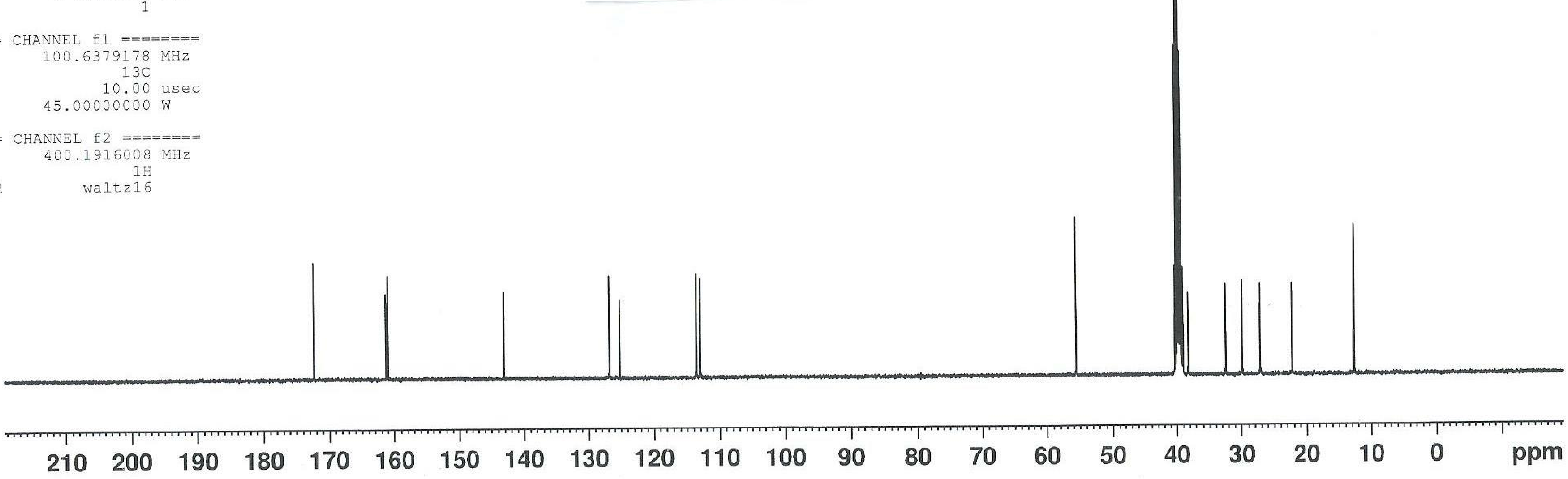

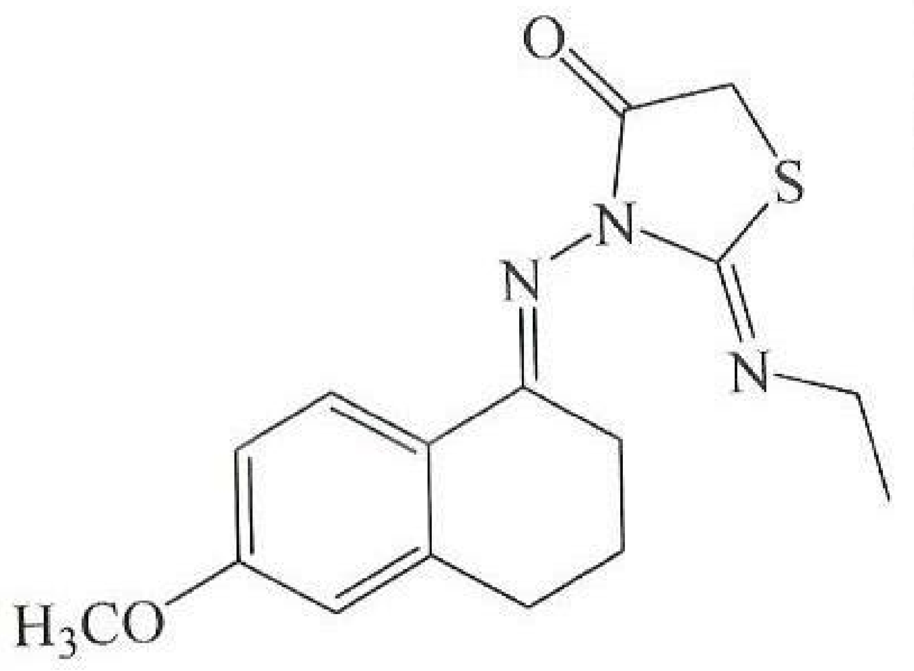

4d

Current Data Parameters
NAME Nesreen Saeed_C_5
EXPNO 10
PROCNO 1

F2 - Acquisition Parameters
Date_ 20190907
Time 11.40
INSTRUM spect
PROBHD 5 mm PABBO BB/
PULPROG zgpg30
TD 65536
SOLVENT DMSO
NS 1200
DS 4
SWH 24038.461 Hz
FIDRES 0.366798 Hz
AQ 1.3631488 sec
RG 202.37
DW 20.800 usec
DE 6.50 usec
TE 298.1 K
D1 2.00000000 sec
D11 0.03000000 sec
TD0 1

======== CHANNEL f1 ========
SFO1 100.6379178 MHz
NUC1 13C
P1 10.00 usec
PLW1 45.00000000 W

======== CHANNEL f2 ========
SFO2 400.1916008 MHz
NUC2 1H
CPDPRG[2 waltz16

10

Microanalytical Unit - FOPCU - NMR laboratory
www.pharma.cu.edu.eg dir-mau.fopcu@pharma.cu.edu.eg

BRUKER

```
Current Data Parameters
NAME       Nesreen Saied_H_I
EXPNO                     50
PROCNO                     1

F2 - Acquisition Parameters
Date_              20191002
Time                  14.39
INSTRUM               spect
PROBHD    5 mm PABBO BB/
PULPROG                zg30
TD                    65536
SOLVENT                DMSO
NS                       32
DS                        2
SWH             8012.820 Hz
FIDRES          0.122266 Hz
AQ             4.0894465 sec
RG                    180.8
DW               62.400 usec
DE                 6.50 usec
TE                  298.1 K
D1           1.00000000 sec
TD0                       1

======== CHANNEL f1 ========
SFO1        400.1924713 MHz
NUC1                     1H
P1                15.00 usec
PLW1        10.39999962 W

F2 - Processing parameters
SI                    65536
SF          400.1900000 MHz
WDW                      EM
SSB        0
LB                  0.30 Hz
GB         0
PC                     1.00
```

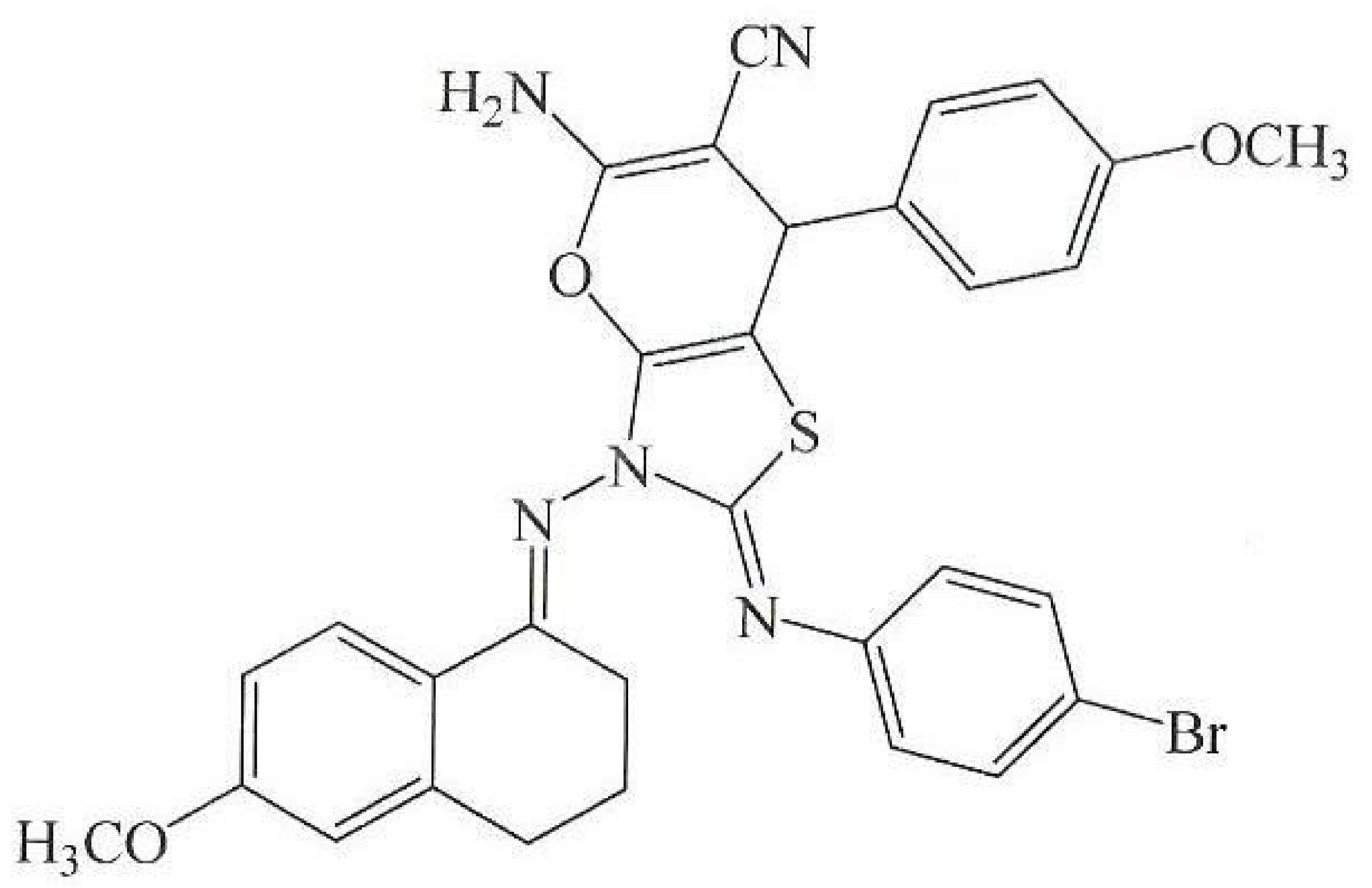

5a

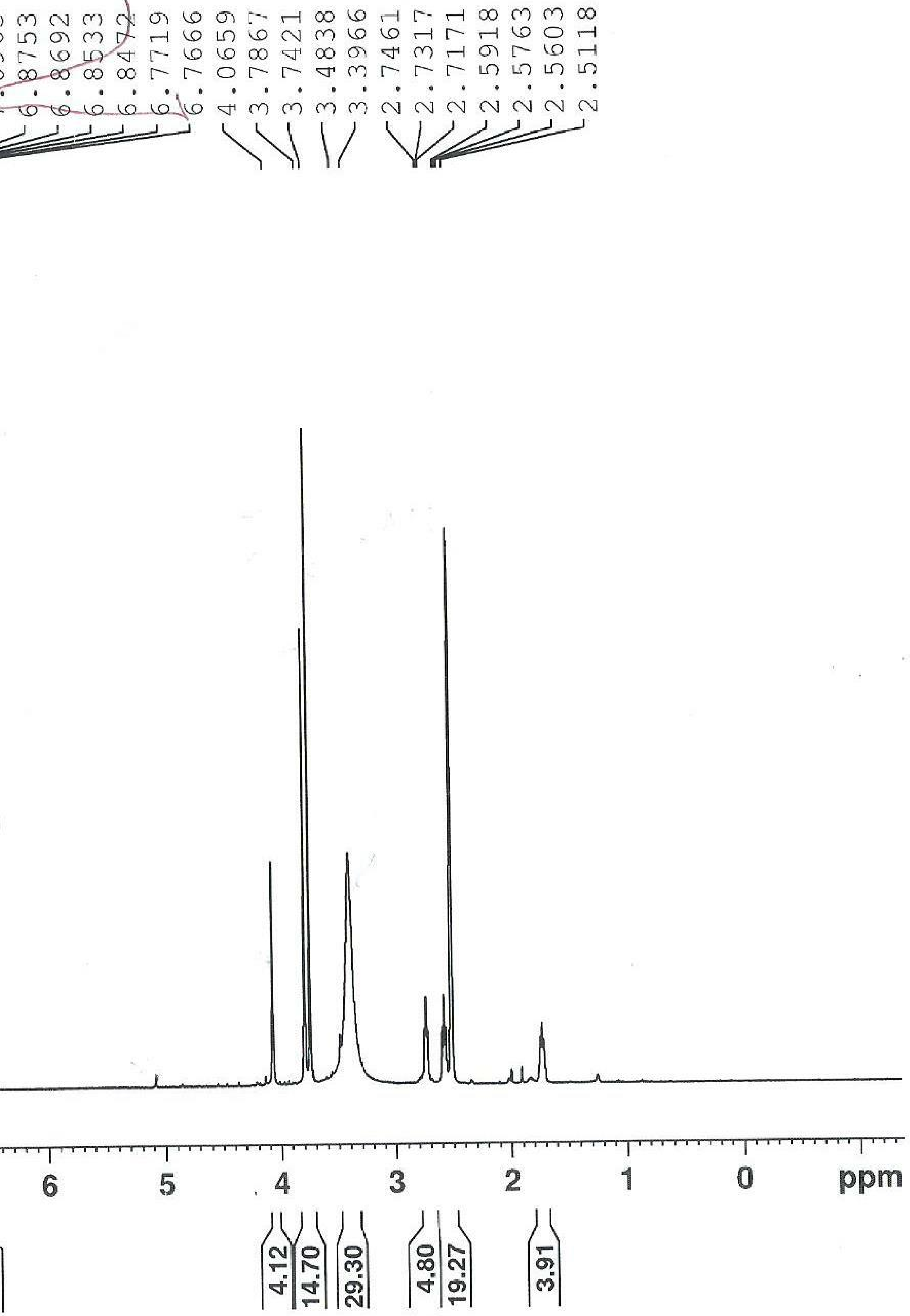

11

Microanalytical Unit - FOPCU - NMR laboratory
www.pharma.cu.edu.eg dir-mau.fopcu@pharma.cu.edu.eg

BRUKER

```
Current Data Parameters
NAME       Nesreen Saeed_C_I
EXPNO                     10
PROCNO                     1

F2 - Acquisition Parameters
Date_               20191030
Time                    2.43
INSTRUM                spect
PROBHD    5 mm PABBO BB/
PULPROG              zgpg30
TD                     65536
SOLVENT                 DMSO
NS                      1200
DS                         4
SWH               24038.461 Hz
FIDRES             0.366798 Hz
AQ                1.3631488 sec
RG                    202.37
DW                    20.800 usec
DE                      6.50 usec
TE                     298.1 K
D1               2.00000000 sec
D11              0.03000000 sec
TD0                        1

======== CHANNEL f1 ========
SFO1           100.6379178 MHz
NUC1                     13C
P1                     10.00 usec
PLW1            45.00000000 W

======== CHANNEL f2 ========
SFO2           400.1916008 MHz
NUC2                      1H
CPDPRG[2             waltz16
```

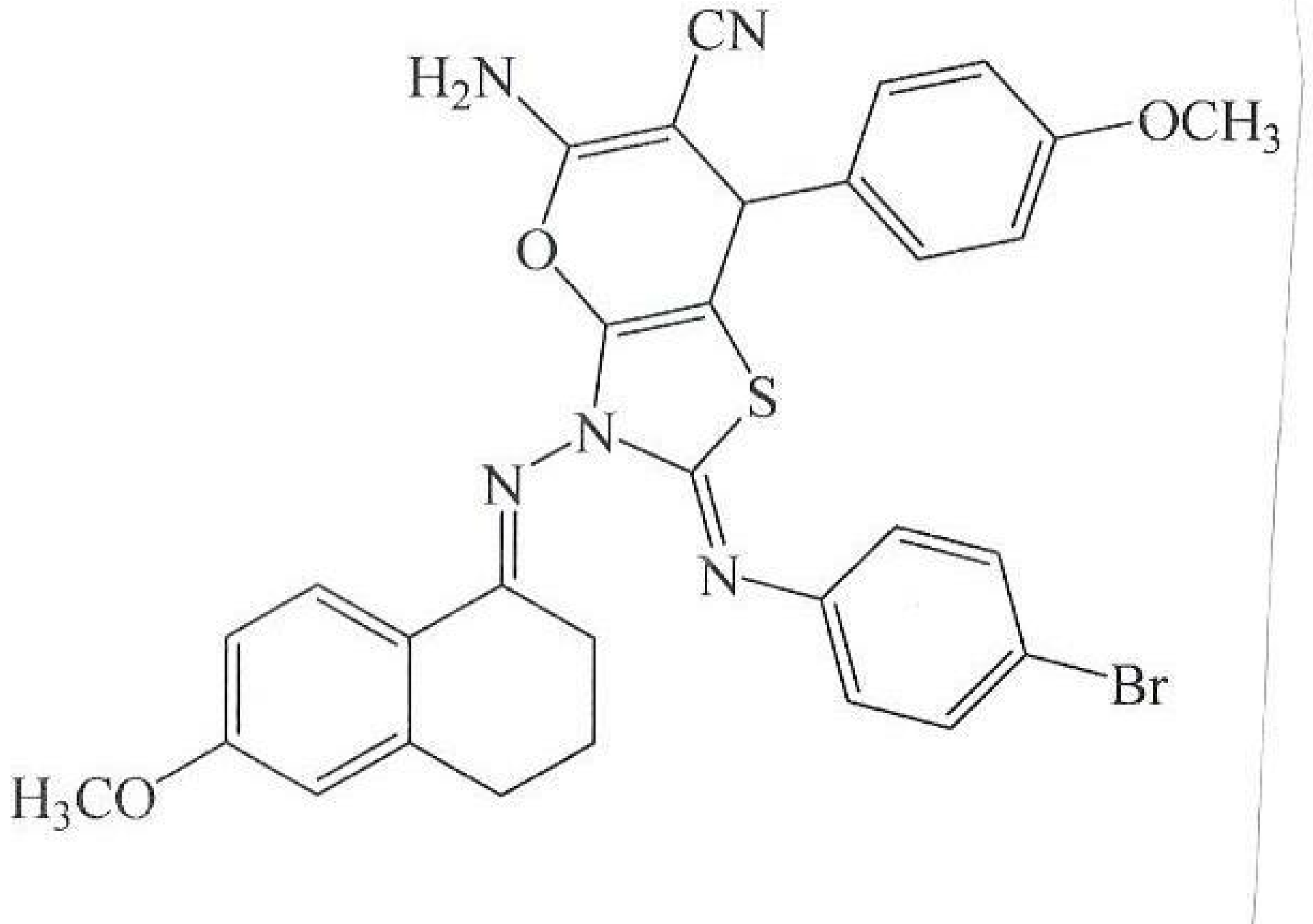

5a

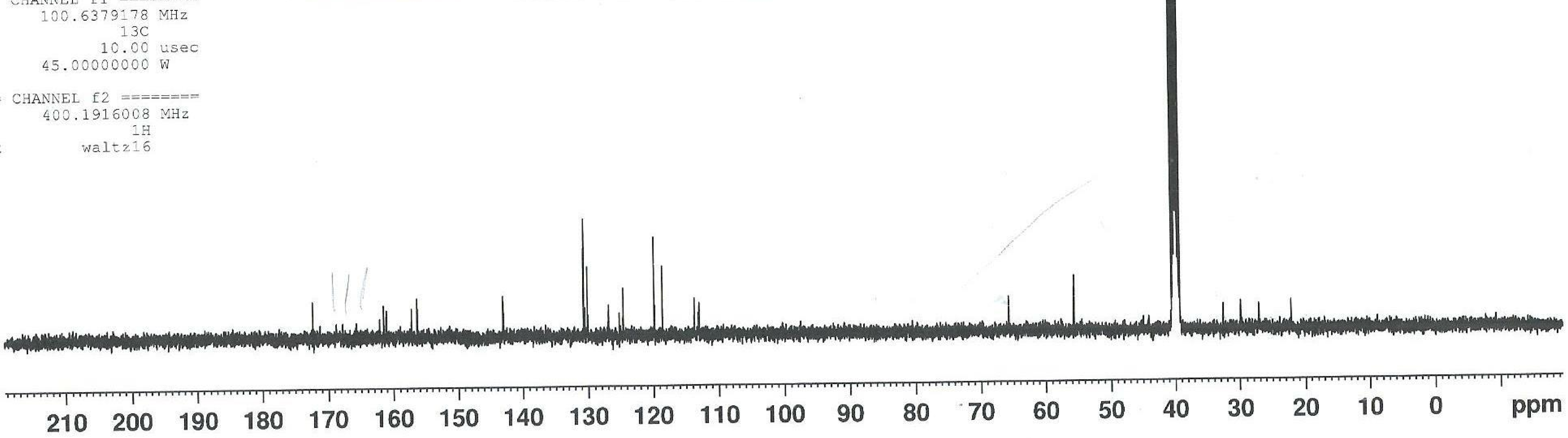

Microanalytical Unit - FOPCU - NMR laboratory
www.pharma.cu.edu.eg dir-mau.fopcu@pharma.cu.edu.eg

BRUKER

Current Data Parameters
NAME Nesreen Saeed_C_9
EXPNO 10
PROCNO 1

F2 - Acquisition Parameters
Date_ 20191030
Time 1.32
INSTRUM spect
PROBHD 5 mm PABBO BB/
PULPROG zgpg30
TD 65536
SOLVENT DMSO
NS 1200
DS 4
SWH 24038.461 Hz
FIDRES 0.366798 Hz
AQ 1.3631488 sec
RG 202.37
DW 20.800 usec
DE 6.50 usec
TE 298.1 K
D1 2.00000000 sec
D11 0.03000000 sec
TD0 1

======== CHANNEL f1 ========
SFO1 100.6379178 MHz
NUC1 13C
P1 10.00 usec
PLW1 45.00000000 W

======== CHANNEL f2 ========
SFO2 400.1916008 MHz
NUC2 1H
CPDPRG[2 waltz16

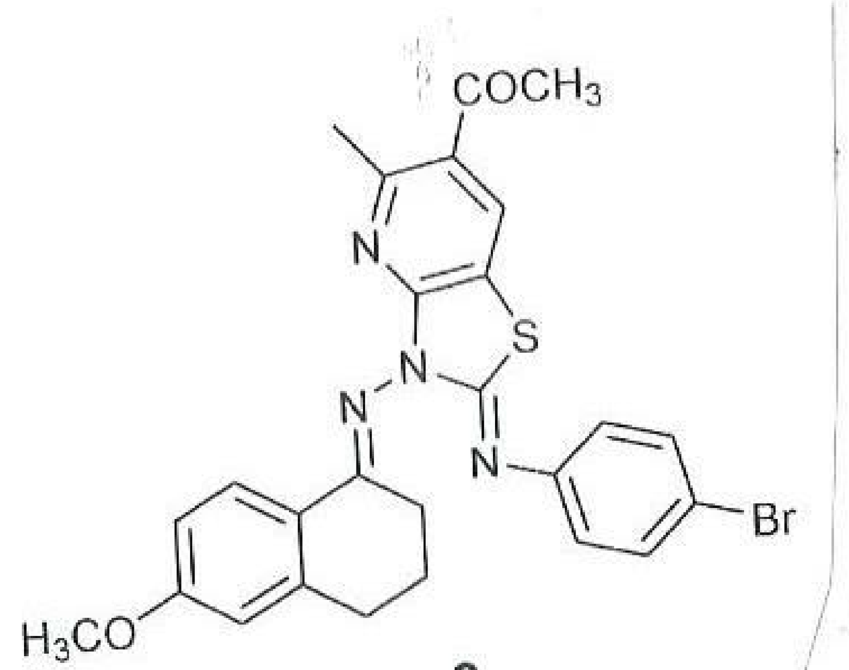

8

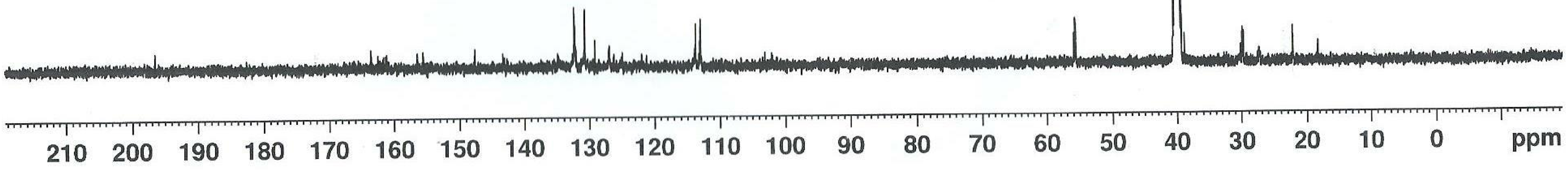

13

Microanalytical Unit - FOPCU - NMR laboratory
www.pharma.cu.edu.eg dir-mau.fopcu@pharma.cu.edu.eg

BRUKER

```
Current Data Parameters
NAME        Nesreen Saeed_C_12
EXPNO                       10
PROCNO                       1

F2 - Acquisition Parameters
Date_                20191030
Time                    10.52
INSTRUM                 spect
PROBHD     5 mm PABBO BB/
PULPROG               zgpg30
TD                     65536
SOLVENT                 DMSO
NS                       200
DS                         4
SWH            24038.461 Hz
FIDRES          0.366798 Hz
AQ             1.3631488 sec
RG                    202.37
DW                20.800 usec
DE                  6.50 usec
TE                   298.0 K
D1            2.00000000 sec
D11           0.03000000 sec
TD0                        1

======== CHANNEL f1 ========
SFO1         100.6379178 MHz
NUC1                     13C
P1                 10.00 usec
PLW1         45.00000000 W

======== CHANNEL f2 ========
SFO2         400.1916008 MHz
NUC2                      1H
CPDPRG[2             waltz16
```

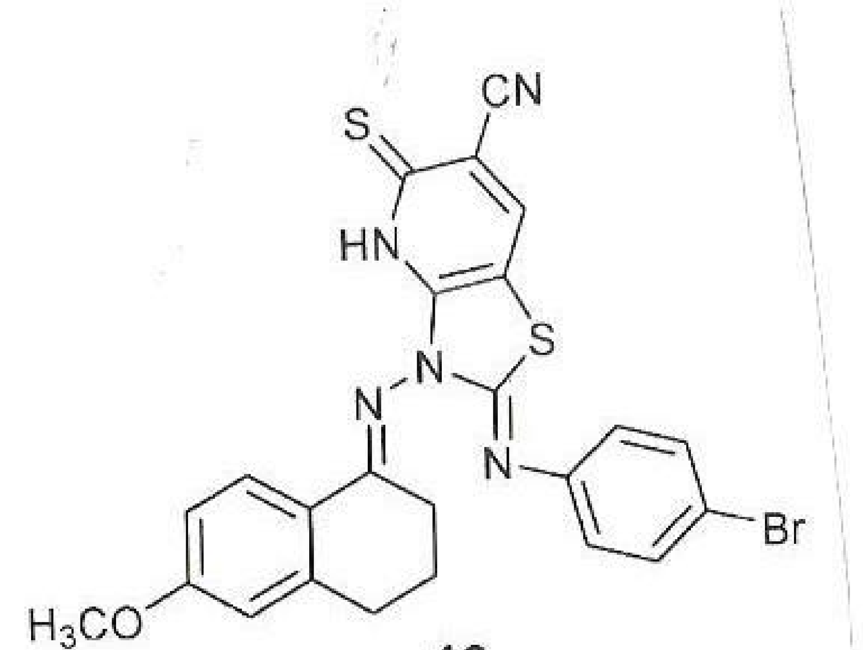

10

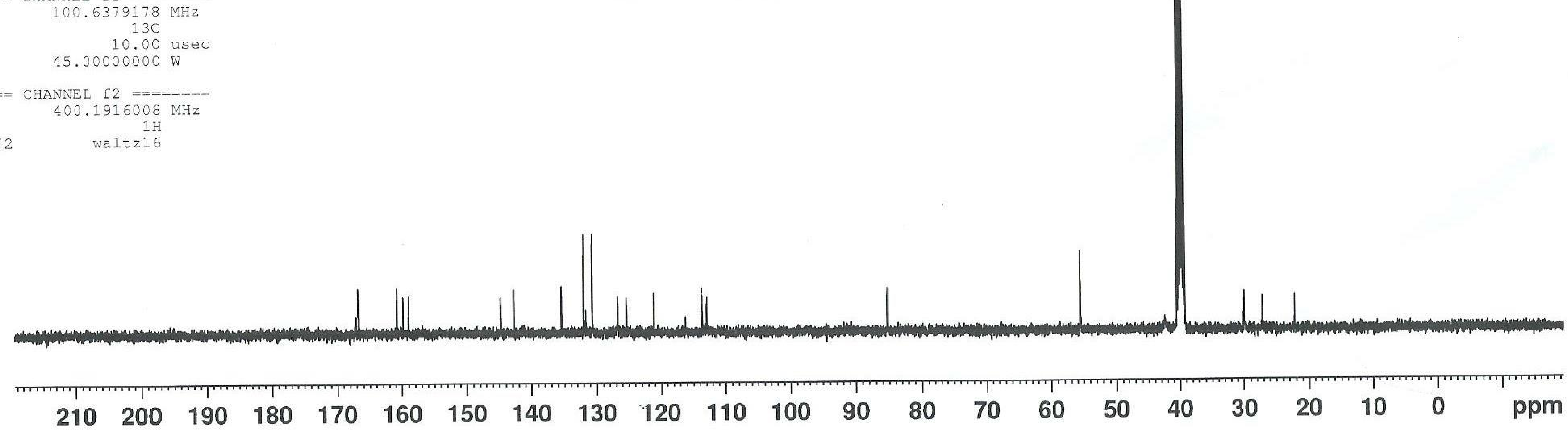

Supplement: Supplementary Materials — The supplementary file includes the Spectroscopic charts which support the study. [file 8649745.f1.pdf]
